# MicroRNA‐204‐5p Deficiency within the vmPFC Region Contributes to Neuroinflammation and Behavioral Disorders via the JAK2/STAT3 Signaling Pathway in Rats

**DOI:** 10.1002/advs.202403080

**Published:** 2025-01-10

**Authors:** Xiao Chen, Yeting Gan, Kaiqi Zhang, Yuhan Wu, Ye Li, Tian Lan, Xianghua Zhuang, Shihong Chen, Shuyan Yu

**Affiliations:** ^1^ Key Laboratory of Mental Disorders The Second Hospital of Shandong University School of Basic Medical Sciences Shandong University Jinan Shandong 250012 China; ^2^ Department of Endocrinology and Metabolism The Second Hospital of Shandong University Jinan Shandong 250033 China; ^3^ Department of Medical Psychology and Ethics School of Basic Medical sciences Cheeloo College of Medicine Shandong University Jinan Shandong 250012 China

**Keywords:** JAK2/STAT3 signaling pathway, MDD, miR‐204‐5p, neuroinflammation, neuronal apoptosis, synaptic plasticity

## Abstract

Major depressive disorder (MDD) is usually considered associate with immune inflammation and synaptic injury within specific brain regions. However, the molecular mechanisms underlying the neural deterioration resulting in depression remain unclear. Here, it is found that miR‐204‐5p is markedly downregulated in the ventromedial prefrontal cortex (vmPFC) in a chronic unpredictable mild stress (CUMS) induce rat model of depression. Knockdown of miR‐204‐5p in the vmPFC of normal rats results in depression and anxiety‐like behaviors accompanied with the activation of microglia, elevated levels of pro‐inflammatory cytokines, and increased numbers of neural apoptotic cells, effects which appear to be mediated by activation of the JAK2/STAT3 signaling pathway. Electrophysiological recordings further demonstrate that knockdown of miR‐204‐5p induces abnormal excitability of pyramidal neurons. In contrast, upregulation of miR‐204‐5p in the vmPFC of CUMS rats significantly causes inhibition of JAK2/STAT3 signaling pathway, improvements in neuronal impairments, and an abolition of the depression and anxiety‐like behaviors. Moreover, pharmacological blocking of the JAK2/STAT3 signaling pathway significantly ameliorates abnormal behaviors resulting from miR‐204‐5p deficiency within the vmPFC. Collectively, these results provide robust evidence that the miR‐204‐5p/JAK2/STAT3 pathway may critically involve in the pathogenesis of depression, which may serve as potentially critical therapeutic target in the treatment of MDD.

## Graphical abstract

The present study shows that miR‐204‐5p deficiency contributes to the enhancement of neuroinflammation and apoptosis which are involved in the pathogenesis of depression‐like behaviors via activating JAK2/STAT3 signaling pathway, while overexpression of miR‐204‐5p significantly ameliorates the neuronal deterioration and behavioral disorders in rats. These results reveal new insights into the neuroprotective effects of miR‐204‐5p which contributes to depression treatment.

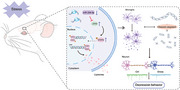

## 1 Introduction

Major depressive disorder (MDD) is a prevalent psychiatric disease that severely limits psychosocial functioning and reduces the quality of life in these patients. MDD typically manifests as a downcast mood, anhedonia, lack of motivation, and cognitive deficits.^[^ ^1^ ^]^ According to reports of the World Health Organization, depression has become the leading cause of mental and physical disability worldwide. Many people diagnosed with MDD are usually characterized by an enduring tendency to experience anxiety, show a poor level of resilience under stress, and usually experience a variety of chronic diseases or other emotional disorders. Such an array of symptoms creates considerable challenges with regard to the clinical detection, diagnosis, and treatment of this condition.^[^ ^2^ ^]^

MicroRNAs (miRNAs) represent endogenous non‐coding single‐stranded RNAs that regulate gene expression at the post‐transcriptional level.^[^ ^3^ ^]^ MiRNAs have been shown to play fundamental roles in many cellular and molecular processes, including neurodevelopment, brain plasticity as well as cell maturation, differentiation, and survival.^[^ ^4^ ^]^ They also play a crucial role in axonal and dendritic outgrowth and morphological maintenance.^[^ ^5^ ^]^ Of particular relevance to this report are miR‐15b,^[^ ^6^ ^]^ miR‐207,^[^ ^7^ ^]^ miR‑9‑5p,^[^ ^8^ ^]^ miR‐124,^[^ ^9^ ^]^ miR‐1202^[^ ^10^ ^]^ and miR‐532,^[^ ^11^ ^]^ which have all been reported play important roles in regulating the development of depression. Accordingly, these studies provide substantial evidence for the participation of miRNAs in depression. In addition, due to the widespread distribution of miRNAs within the central nervous system, emerging evidence has accrued indicating that miRNAs play crucial and widespread roles in neuropathology, especially in processes involved with neuroinflammation and apoptosis.^[^ ^12^ ^]^ For example, by targeting Cxcl12, miR‐874‐3p restrained neuroinflammation, while the knockdown of miR‐20b protected neurons from inflammatory responses and oxidative stress damage.^[^ ^13^ ^]^

MicroRNA‐204‐5p (miR‐204‐5p) is one of most abundant miRNAs in the brain. Findings from a number of reports have indicated that miR‐204‐5p was closely related to the occurrence of many diseases, and it shows considerable variations in its expression and roles as related to different diseases.^[^ ^14^ ^]^ In a study of sensorineural hearing loss (SNHL), miR‐204‐5p was suggested as being involved in the functional recovery of impaired spiral ganglion neurons.^[^ ^15^ ^]^ In addition, miR‐204‐5p ameliorates neurological injury in cerebral ischemia reperfusion injury, and ischemic stroke.^[^ ^16^ ^]^ A reduction of miR‐204‐5p within the rat hippocampus contributes to a stress‐induced pathology as a result of targeting the RGS12 signaling pathway.^[^ ^17^ ^]^ Collectively, these studies all indicate a neuroprotective role of miR‐204‐5p. Interestingly, findings from a recent survey have indicated that autophagy was mitigated by miR‐204‐5p in middle cerebral artery occlusion (MCAO) rats.^[^ ^18^ ^]^ Although the role of miR‐204‐5p in neuroprotection and autophagy has been studied, its potential as related to the neuroinflammation and apoptosis as associated with depression has yet to be investigated.

Janus kinase 2 (JAK2)/signal transduction and activator of transcription 3 (STAT3) represent signal transduction pathways localized to the cytoplasm. Cytokine receptors activate JAK2 phosphorylation, followed by an activation of cytoplasmic STAT3 which then translocates to the nucleus to control the transcription of targeted downstream genes.^[^ ^19^ ^]^ The JAK2/STAT3 pathway plays a critical role in inflammatory reactions, which are necessary for the function of cytokines, the differentiation of T lymphocytes, and the activation of adaptive immunity.^[^ ^20^ ^]^ Increasing evidence has accrued demonstrating that the JAK2/STAT3 signaling pathway plays a pivotal role in microglia activation, and acts as a potential therapeutic flashpoint in neuroinflammation and neuronal apoptosis.^[^ ^21^ ^]^ Based on the results from these previous studies, we considered the role and importance that both miR204‐5p and the JAK2/STAT3 signaling pathway play as associated with neuroinflammation and apoptosis. However, their potential relationship has yet to be investigated. As based on bioinformatics analysis, JAK2 is predicted to be a candidate gene of miR‐204‐5p. Thus, we hypothesized that miR‐204‐5p may regulate neuroinflammation and apoptosis via the JAK2/STAT3 signaling pathway, a relationship which may then be targeted for use in alleviating depression.

Therefore, in this study, with use of the chronic unpredictable mild stress (CUMS)‐induced rat model of depression, we investigated the effects of alterations in the expression of miR‐204‐5p within the ventromedial prefrontal cortex (vmPFC) region. As based on the protein–protein interaction (PPI) network analysis and protein docking, JAK2 has been identified as interacting with the STAT3 protein. Here, we found that phosphorylation of the JAK2/STAT3 signaling pathway was upregulated within the vmPFC after knockdown of miR‐204‐5p. In contrast, with an upregulation of miR‐204‐5p within the vmPFC region of CUMS rats, the display of depression‐ and anxiety‐like phenotypes was significantly ameliorated. In summary, these results provide important, new insights into the mechanisms of the miR‐204‐5p/ JAK2/STAT3 signaling pathway that may be involved with depression and may thus serve as a potential new target for the prognosis and treatment of this condition.

## 2 Results

### 2.1 CUMS Produces Depressive‐ and Anxiety‐Like Behaviors and Impairs Synaptic Plasticity within the vmPFC Region

**Figure 1:**
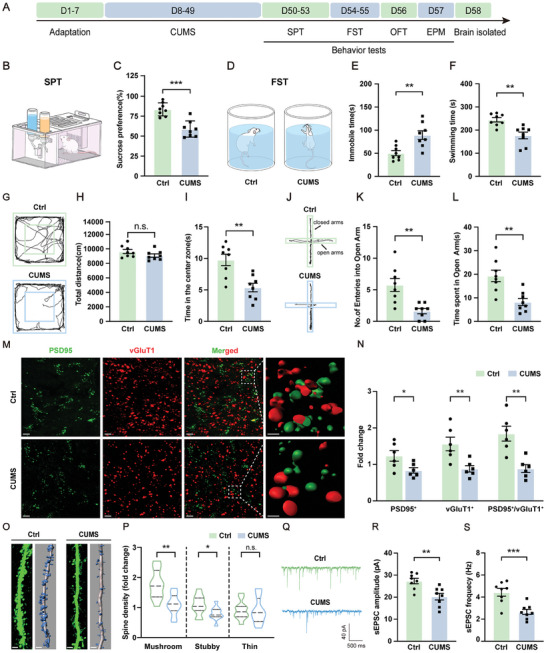
CUMS exposure decreased synaptic density and damaged synaptic plasticity within the vmPFC region in rats. A) Schematic showing the CUMS protocol used to induce depressive‐like behaviors in rats. B,C) SPT results show that sucrose preference was decreased in CUMS rats. D–F) FST results show that immobility times were increased and swimming times decreased in CUMS rats. G–I) Results of OFT. G) Raw traces of locomotor activity in the OFT; H) total distance traveled in the OFT did not significantly differ between Ctrl and CUMS rats; I) time spent exploring the center area in the OFT was decreased in CUMS rats. J–L) Results of EPM. J) Raw traces of movements in the EPM; K) the number of open arm entries was decreased in CUMS rats; L) time spent in the open arms was decreased in CUMS rats (*n* = 8 rats per group for behavior tests). M) 3D rendering of confocal stacks showing PSD95 (green) and vGluT1 (red) puncta in the vmPFC region. 3D reconstruction of zoom‐in images indicated colocalized pre‐ and post‐synaptic puncta. Scale bar, 5 µm. Zoom‐in images with a scale bar equal to 15 µm. N) CUMS exposure decreased PSD95 puncta density, vGluT1 puncta density, and PSD95‐vGluT1 colocalization density (*n* = 6 rats per group). O) Representative dendrites from the vmPFC region showing sparse labeling and 3D reconstruction of eGFP. Scale bar, 5 µm. P) CUMS decreased the density of the three types of dendritic spines (*n* = 3–4 rats per group, 10–12 dendrites per group were counted). Q–S) Results of sEPSC. Q) Raw traces of sEPSC in vmPFC pyramidal neurons. Scale bars = 500 ms, 40 pA. R) CUMS exposure decreased the amplitude of sEPSC. S) CUMS exposure decreased the frequency of sEPSC. n.s., not significant, *p* > 0.05, *p < 0.05, **p < 0.01, ***p < 0.001, by an unpaired two‐tailed Student's *t*‐test (C, E, F, H, I, K, L, R, S) and a two‐way ANOVA (N, P), data represent means ± SEMs. Ctrl, control.

A CUMS regimen was used to establish an animal model of depression which was then assessed using four different behavioral tests (**Figure** **1**A). In the sucrose preference test (SPT), CUMS rats displayed a significantly reduced percent of sucrose consumption compared with control rats (Figure 1B,C), while in the forced swimming test (FST), these rats demonstrated increased immobility times and decreased swimming times (Figure 1D–F). The results of these two tests suggest that CUMS rats displayed anhedonia and behavioral despair, which are typical depression‐like behaviors. Results from the open field test (OFT) showed that CUMS rats exhibited a decrease in time exploring the center area, while their total distance traveled did not differ from that of controls (Figure 1G–I). Finally, in the elevated plus maze (EPM) test, CUMS rats spent significantly less time and fewer entries into the open arms (Figure 1J–L). The results of these latter two tests suggest that CUMS exposure increased the display of depression‐related fear and anxiety.

To determine whether this CUMS treatment led to potential synaptic changes, we performed a double immunofluorescent staining for PSD95 and the vesicular glutamate transporter 1 (vGluT1). The number of puncta for both PSD95 and vGluT1 was significantly decreased in CUMS rats. Moreover, the number of functional synaptic contacts, as indicated by vGluT1‐PSD95 colocalizations in zoom images of 3D reconstructions was significantly reduced in the vmPFC of CUMS rats (Figure 1M,N). In addition, we quantified the changes in densities of three types of dendritic spines (mushroom, stubby, and thin) in the vmPFC region using Sparse labeling and Golgi staining. Typically, stubby and thin spines are considered as immature subtypes, while mushroom spines represent a more mature and stable subtype. Our results showed that CUMS exposure decreased the number of dendritic mushroom spines (Figure 1O,P; Figure S1, Supporting Information). As CUMS exposure is associated with structural synaptic plasticity damage, we then examined functional synaptic plasticity by recording spontaneous excitatory postsynaptic currents (sEPSCs). Slices from CUMS rats exhibited significant impairments in the amplitudes and frequencies of sEPSC in pyramidal neurons (Figure 1Q–S). These findings suggest that chronic stress impaired synaptic plasticity in vmPFC pyramidal neurons, which may then contribute to the depressive‐ and anxiety‐like behaviors observed in these CUMS rats.

### 2.2 Knockdown of miR‐204‐5p within the vmPFC Region Induces Depressive‐ and Anxiety‐Like Behaviors

**Figure 2:**
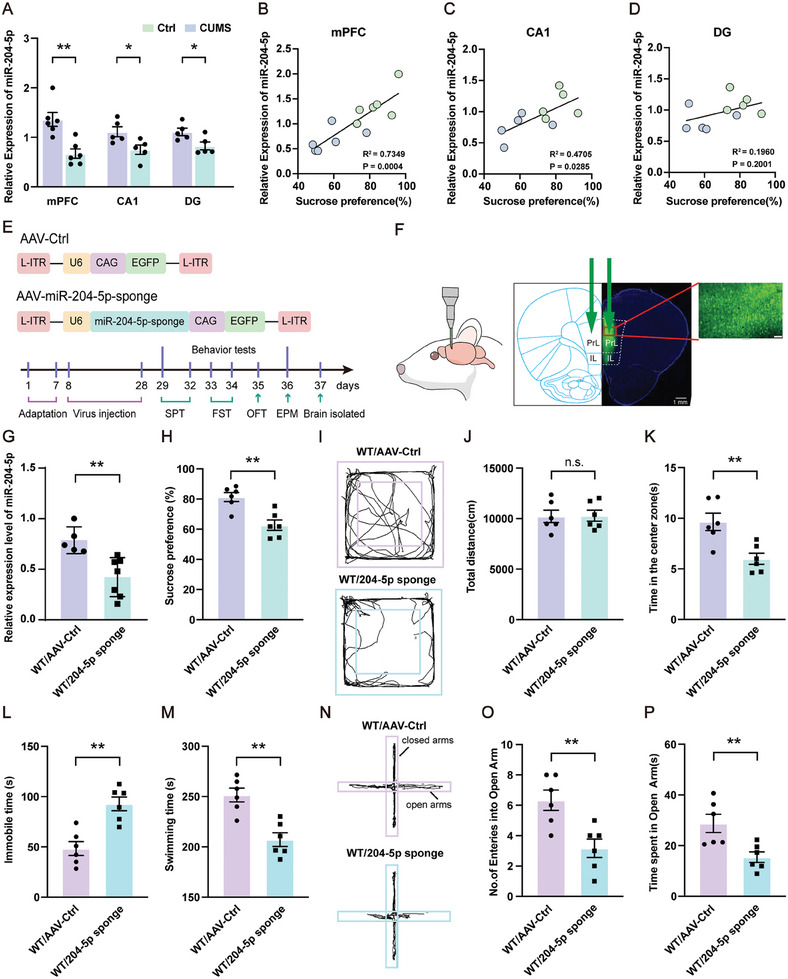
Chronic stress decreased miR‐204‐5p expression and a miR‐204‐5p deficiency produced depression‐like behaviors in normal rats. A) The mRNA expression of miR‐204‐5p within the vmPFC, CA1, and DG regions (N = 5–6 rats per group). Correlations between sucrose preference scores and miR‐204‐5p levels within the B) vmPFC, C) CA1 region, and D) DG region (*n* = 5–6 rats per group). E) Schematic of experimental design and AAV vectors engineered to knockdown miR‐204‐5p or a vector control construct. F) Illustration of bilateral virus injection sites into the vmPFC region. Scale bar: 88 1 mm. Zoom‐in images with a scale bar equal to 100 µm. G) qPCR results showing knockdown efficiency of miR‐204‐5p in the vmPFC region (*n* = 5–7 rats per group). H) Knockdown of miR‐204‐5p in the vmPFC region decreased sucrose preference. I–K) OFT results. I) Raw traces of locomotor activity in the OFT; J) total distance traveled in the OFT was not significantly different between WT/AAV‐Ctrl and WT/204‐5p sponge rats; K) time spent exploring the center area in the OFT was decreased in miR‐204‐5p knockdown rats. L–M) FST results. L) Immobility times were increased in miR‐204‐5p knockdown rats; M) swimming times were decreased in miR‐204‐5p knockdown rats. N–P) EPM results. N) Raw traces of movements in the EPM; O) the number of open arm entries was decreased in miR‐204‐5p knockdown rats; P) time spent in the open arms was decreased in miR‐204‐5p knockdown rats (*n* = 6 rats per group for behavior tests). n.s., not significant, *p* > 0.05, *p < 0.05, **p < 0.01, ***p < 0.001, by a two‐way ANOVA(A), one‐way ANOVA with Bonferroni's multiple‐comparison test B, C, D) and unpaired two‐tailed Student's *t*‐test G, H, J, K, L, M, O, P), data represent means ± SEMs.

Results from previous studies have indicated that miR‐204‐5p is associated with a variety of nervous system related diseases.^[^ ^15^, ^16^, ^17^, ^22^ ^]^ As an approach to assess the antidepressant and anxiolytic effects of miR‐204‐5p and the underlying mechanisms, we examined the expression of miR‐204‐5p in CUMS rats with use of qPCR assays. We found that miR‐204‐5p was significantly decreased within the vmPFC region and the anhedonia symptoms observed in these rats were positively correlated with miR‐204‐5p levels. Interestingly, this effect appears to be specific for the vmPFC, as no such decreases were evident in the hippocampus CA1 and DG regions (**Figure** **2**A–D). In addition, we cultured neurons, primary microglia, and astroglial cells to determine whether miR‐204‐5p expression changes in different types of cells within the brain. Our qPCR analyses showed that miR‐204‐5p was highly expressed and more sensitive to lipopolysaccharide (LPS) stimulation in neurons versus that observed in microglia and astroglia (Figure S2, Supporting Information). To investigate whether a reduction in miR‐204‐5p expression within the vmPFC was associated with depressive‐ and anxiety‐like behaviors in rats, we constructed an adeno‐associated virus (AAV) vector (AAV‐miR‐204‐5p sponge) engineered to knock down miR‐204‐5p via a bilateral injection into the vmPFC of normal rats, while another vector including a scrambled sequence was constructed to serve as a negative control (Figure 2E,F). Three weeks after virus injection, the knockdown efficiency of miR‐204‐5p was assessed using qPCR (Figure 2G). Behaviorally, the knockdown of miR‐204‐5p produced a reduction in sucrose preference in the SPT (Figure 2H) and increased immobility times, and decreased swimming times in the FST (Figure 2L,M). In the OFT, this miR‐204‐5p deficiency produced a decrease in time exploring the center area, but no effect on the total amount of spontaneous locomotor activity in these rats (Figure 2I–K). Results from the EPM showed that miR‐204‐5p knockdown rats spent significantly less time and entries into the open arms as compared to control rats (Figure 2N–P). These results demonstrate that rats develop depressive‐ and anxiety‐like phenotypes in response to the knockdown of miR‐204‐5p within the vmPFC. Accordingly, a deficiency of miR‐204‐5p at this site may be a potential risk factor for depression and anxiety.

### 2.3 MiR‐204‐5p Directly Targets the JAK2/ STAT3 Signaling Pathway

**Figure 3:**
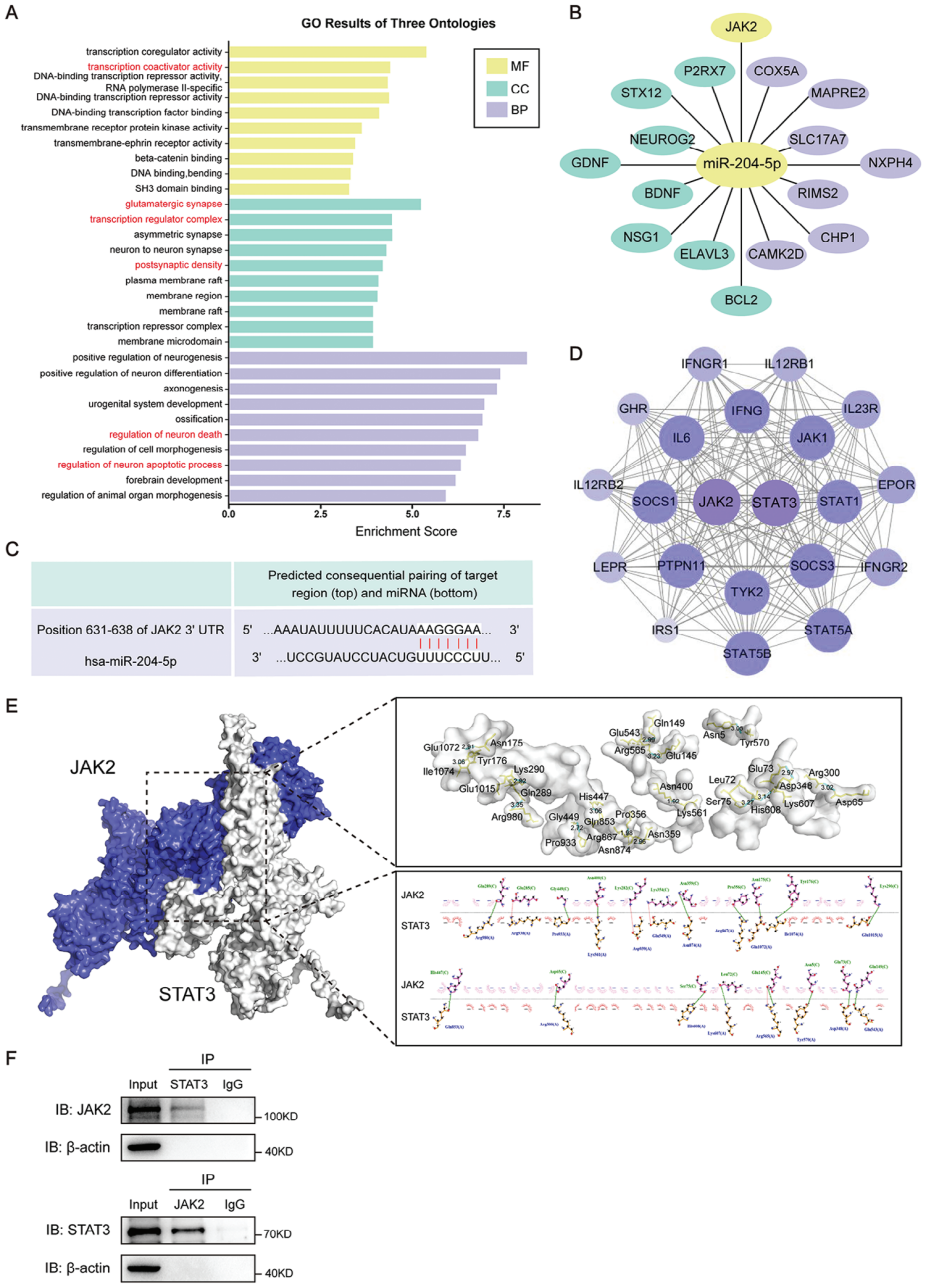
MiR‐204‐5p directly targeted the JAK2/STAT3 signaling pathway. A) GO results of three ontologies. Bar graphs represent magnitudes of significant correlations for miR‐204‐5p mediated signaling pathways. B) Bioinformatical prediction of miR‐204‐5p target genes. C) Predicted putative seed‐matching sites between miR‐204‐5p and JAK2. D) The protein–protein interaction (PPI) network of JAK2 and STAT3. E) The possible interaction mode of JAK2 and STAT3 derived from rats. F) Representative Western blots of the co‐immunoprecipitation of JAK2 and STAT3.

To identify potential targets for miR‐204‐5p, we considered some of the downstream target genes of miR‐204‐5p as contained in the target gene prediction website Targetscan 8.0. With this approach, a total of 791 potential target genes were identified. A gene ontology (GO) enrichment analysis was then performed on these potential target genes, with the results revealing that target genes of miR‐204‐5p were found to be associated with multiple signaling pathways, including transcription activators and neuronal apoptosis (**Figure** **3**A; Figure S3, Supporting Information). Among the predicted target genes regulated by miR‐204‐5p, we focused on JAK2, which contributes to neuroinflammation and apoptosis, and it was predicted that the 3′‐UTR of JAK2 mRNA would contain a putative binding site for the seed‐match sequence of miR‐204‐5p (Figure 3B,C). Moreover, the protein–protein interaction (PPI) network analysis showed that there may exist a direct interaction between STAT3, which mediates the expression of several apoptosis‐related genes, and the JAK2 protein (Figure 3D). The protein docking as observed between these proteins suggested the possibility of an interaction between JAK2 and STAT3 as derived from rats (Figure 3E). The left figure of Figure 3E shows the interaction mode diagram of JAK2 and STAT3, while the two figures on the right show the interactions across the interface in 3‐ (3D) and 2‐ (2D) directions, respectively. It can be seen that the amino acid residues Gln289, Glu285, Gly449… of JAK2 form 16 hydrogen bonds with Arg980, Arg938, Pro933… residues of STAT3 respectively, and that hydrophobic interactions may be formed between other residues as shown in this figure. From co‐immunoprecipitation analysis, we further verified that STAT3 is a molecular target of JAK2, as JAK2 demonstrated a definite interaction with STAT3 (Figure 3F). Therefore, these results indicate that miR‐204‐5p may directly target the JAK2/STAT3 signaling pathway.

### 2.4 MiR‑204‐5p Deficiency Promotes Inflammatory Responses within the vmPFC Region in Rats

**Figure 4:**
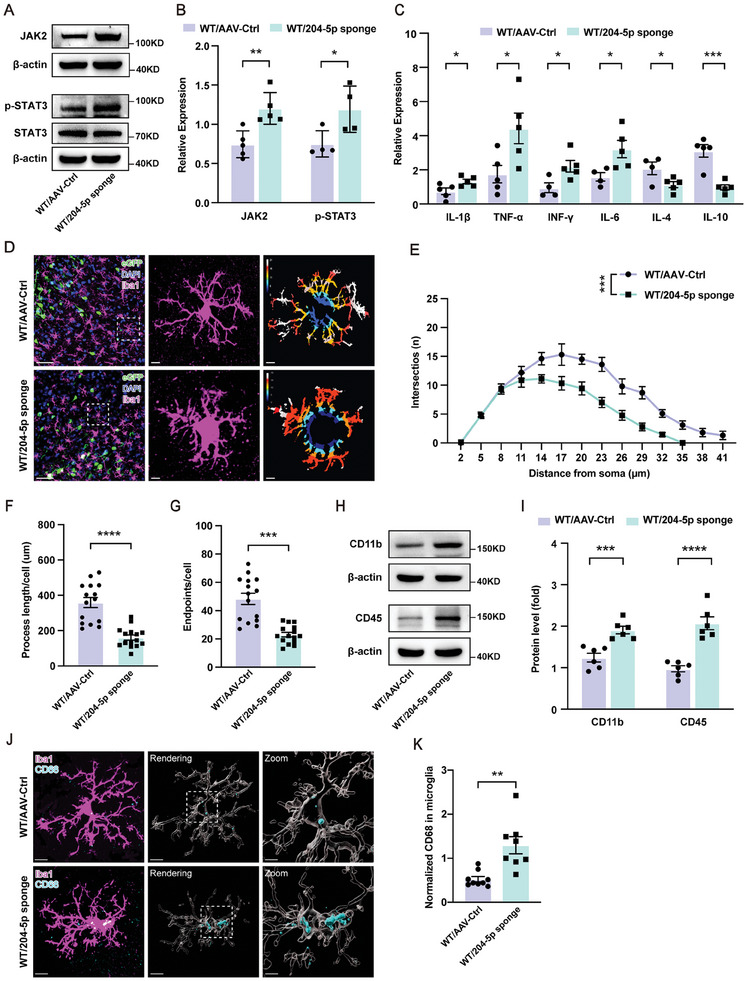
MiR‑204‐5p deficiency promoted inflammatory responses within the vmPFC region. A,B) Protein levels of JAK2 and p‐STAT3 were increased in miR‐204‐5p knockdown rats (*n* = 4–5 rats per group). C) Pro‐inflammatory cytokines were increased and anti‐inflammatory cytokines were decreased in miR‐204‐5p knockdown rats. D) Coronal vmPFC sections stained with DAPI (blue) for nuclei, eGFP (green) for pyramidal neurons, and Iba1 (magenta) for microglia. Representative images of microglia and heat maps of Sholl analysis. Original magnification × 20; scale bar, 40 µm. Zoom‐in images with a scale bar equal to 15 µm. Heat maps with red indicate the radius with the highest number of intersections. E) Sholl analysis showing the number of intersections at different distances from the microglial soma in the indicated groups. F) Process length of microglia within the vmPFC region was significantly decreased in miR‐204‐5p knockdown rats. G) Endpoints of microglia were significantly decreased in miR‐204‐5p knockdown rats (*n* = 4–5 rats per group). H–I) Expressions of CD11b and CD45 were significantly increased in miR‐204‐5p knockdown rats (*n* = 6 rats per group). J,K) Representative images and 3D surface renderings of Iba1^+^ microglia, quantitation of CD68 in the indicated groups (*n* = 4–5 rats per group). Scale bars, 5 and 2 µm. *p < 0.05, **p < 0.01, ***p < 0.001, ****p < 0.0001, by an unpaired two‐tailed Student's *t*‐test F, G, and K), a one‐way ANOVA E) and a two‐way ANOVA B, C, and I), data represent means ± SEMs.

Depression is usually accompanied with neuroinflammation and neuronal apoptosis within specific brain regions. We found that protein levels of JAK2 and p‐STAT3 were significantly increased within the vmPFC of miR‐204‐5p knockdown rats, which indicated that the JAK2/STAT3 signaling pathway was activated in miR‐204‐5p knockdown rats as well as in CUMS rats (**Figure** **4**A,B; Figure S4A,B, Supporting Information). As JAK2‐STAT3 signaling activation occurs in parallel with microglial activation and the release of pro‐inflammatory factors, this pathway represents an attractive molecular target for treating diversified neuroinflammatory‐related diseases.^[^ ^23^ ^]^ Based on these series of events, we next determined mRNA expressions of several critical pro‐inflammatory cytokines, such as interleukin‐1β (IL‐1β), tumor necrosis factor‐α (TNF‐α), interferon gamma (IFN‐γ) and IL‐6 within the vmPFC region and found that all were significantly increased, whereas the anti‐inflammatory cytokines IL‐4 and IL‐10 were significantly decreased after knockdown of miR‐204‐5p (Figure 4C). As the morphology of microglia is closely linked to their function and activation status, immunofluorescent staining was used to examine this morphology with the results showing that a reduction in intersections (Figure 4D,E), shorter processes (Figure 4F) and endpoints (Figure 4G) of microglia were present within the vmPFC region of the WT/204‐5p sponge group as compared to that observed in the control group. Consistently, protein expressions of CD11b and CD45 were increased after knockdown of miR‐204‐5p (Figure 4H,I). Additionally, the levels of CD68 also increased in WT/204‐5p sponge rats associated with microglial activation (Figure 4J,K). These results suggested that a miR‐204‐5p deficiency promoted inflammatory responses within the vmPFC in rats.

### 2.5 Knockdown of miR‐204‐5p within the vmPFC Region Produces Neuronal Apoptosis in Rats

**Figure 5:**
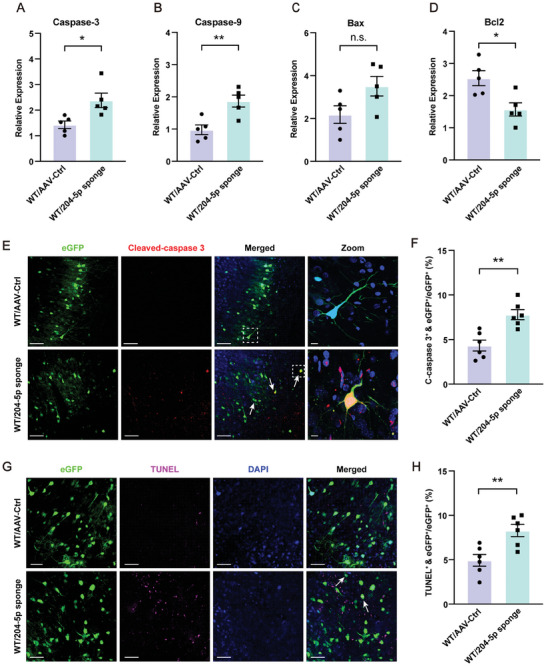
Knockdown of miR‐204‐5p within the vmPFC region produced neuronal apoptosis in rats. A–C) Pro‐apoptotic cytokines were increased in miR‐204‐5p knockdown rats. D) Anti‐apoptotic cytokines were decreased in miR‐204‐5p knockdown rats (*n* = 5 rats per group). E) Immunofluorescent staining showing the number of Cleaved‐Caspase 3 positive cells (red) and eGFP colocalization (White arrows). Scale bar, 40 µm. Zoom‐in images with a scale bar equal to 15 µm. F) Quantification of the number of Cleaved‐Caspase 3 positive cells (red) and eGFP colocalization (*n* = 6 rats per group). G) TUNEL staining. H) Quantification of the number of apoptotic neurons (magenta) and eGFP colocalization (*n* = 6 rats per group). Scale bar, 40 µm. n.s., not significant, *p* > 0.05, *p < 0.05, **p < 0.01, by unpaired two‐tailed Student's *t*‐test, data represent means ± SEMs.

To investigate whether a miR‐204‐5p deficiency was associated with apoptosis, we examined the expression of critical apoptosis‐related cytokines with use of qPCR assays. There was an upregulation of pro‐apoptotic cytokines, such as Caspase‐3, Caspase‐9, and Bax, and a downregulation of the anti‐apoptotic protein, Bcl2, in rats with a knockdown of miR‐204‐5p (**Figure** **5**A–D). Immunofluorescent staining results revealed that Cleaved‐caspase 3 positive cells, terminal regulators that prompt apoptotic processes, were significantly increased in the miR‐204‐5p knock‐down rats (Figure 5E,F). Moreover, the rate of apoptosis was significantly increased within the vmPFC region after knockdown of miR‐204‐5p, as demonstrated in TUNEL assays (Figure 5G,H) as well as in Hoechst staining (Figure S5A, Supporting Information). These results provide robust evidence that downregulation of miR‐204‐5p within the vmPFC contributes to neuronal apoptosis.

### 2.6 Overexpression of miR‐204‐5p within the vmPFC Region Rescued Depressive‐ and Anxiety‐Like Behaviors in CUMS Rats

**Figure 6:**
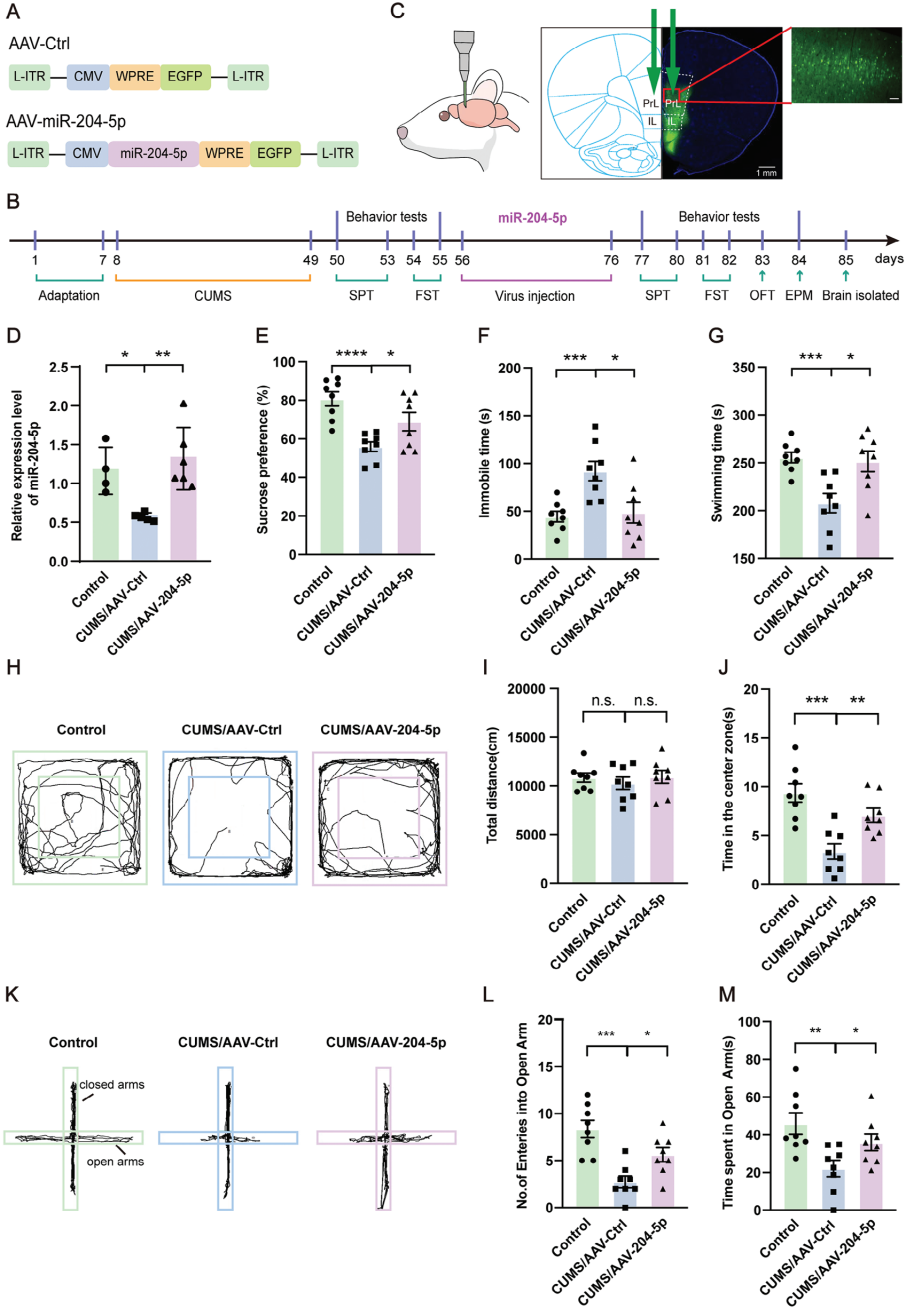
Overexpression of miR‐204‐5p within the vmPFC region rescued depression‐like behaviors in rats. A) Schematic of AAV vectors engineered to overexpress miR‐204‐5p or a vector control construct. B) Experimental design. C) Illustration of bilateral virus injection sites into the vmPFC region. Scale bar: 1 mm. Zoom‐in images with a scale bar equal to 100 µm. D) QPCR results showing overexpression efficiency of miR‐204‐5p within the vmPFC region of CUMS/AAV‐204‐5p rats (*n* = 4–6 rats per group). E) Overexpression of miR‐204‐5p within the vmPFC region increased sucrose preference in CUMS/AAV‐204‐5p rats. F,G) Results of FST. F. Overexpression of miR‐204‐5p within the vmPFC region decreased immobility times in CUMS/AAV‐204‐5p rats; G) overexpression of miR‐204‐5p within the vmPFC region increased swimming times in CUMS/AAV‐204‐5p rats. H–J) Results of OFT. H) Raw traces of locomotor activity in the OFT; I) total distance traveled in the OFT was not significantly different among the three groups; J) overexpression of miR‐204‐5p within the vmPFC region increased the time spent exploring the center area in CUMS/AAV‐204‐5p rats. K–M) Results of EPM. K) Raw traces of movements in the EPM; L) overexpression of miR‐204‐5p within the vmPFC region increased the number of open arm entries in CUMS/AAV‐204‐5p rats; M) Overexpression of miR‐204‐5p within the vmPFC region increased the time spent in the open arms in CUMS/AAV‐204‐5p rats (*n* = 8 rats per group for behavior tests). n.s., not significant, *p* > 0.05, *p < 0.05, **p < 0.01, ***p < 0.001, ****p < 0.0001, by unpaired two‐tailed Student's *t*‐test, data represent means ± SEMs.

As a means to determine whether an overexpression of miR‐204‐5p within CUMS rats would alleviate their depressive/anxiety behavioral responses, we bilaterally infused AAV‐miR‐204‐5p into the vmPFC region of CUMS rats to overexpress miR‐204‐5p (**Figure** **6**A–C) and assessed the efficiency of this overexpression with use of qPCR assays (Figure 6D). When tested at 3 weeks after induction of this miR‐204‐5p overexpression within the vmPFC region we observed a significant reduction in the anhedonia and behavioral despair resulting from CUMS exposure, as evidenced by increased sucrose preferences in the SPT (Figure 6E) and decreased immobility times and increased swimming times in the FST (Figure 6F,G) as compared with CUMS rats receiving the mock virus infusion. In addition, results from the OFT showed that overexpression of miR‐204‐5p increased the time exploring in the center area, but did not alter spontaneous locomotor activity (Figure 6H–J). Finally, with regard to the EPM test, these CUMS rats with overexpression of miR‐204‐5p showed increased times and entries into the open arms of the maze (Figure 6K–M). These findings demonstrate that an upregulation of miR‐204‐5p within the vmPFC of CUMS rats rescues the core symptoms of depression and anxiety displayed by these rats.

### 2.7 Overexpression of miR‐204‐5p Alleviated Inflammatory Responses and Neuronal Apoptosis within the vmPFC Region in CUMS Rats

**Figure 7:**
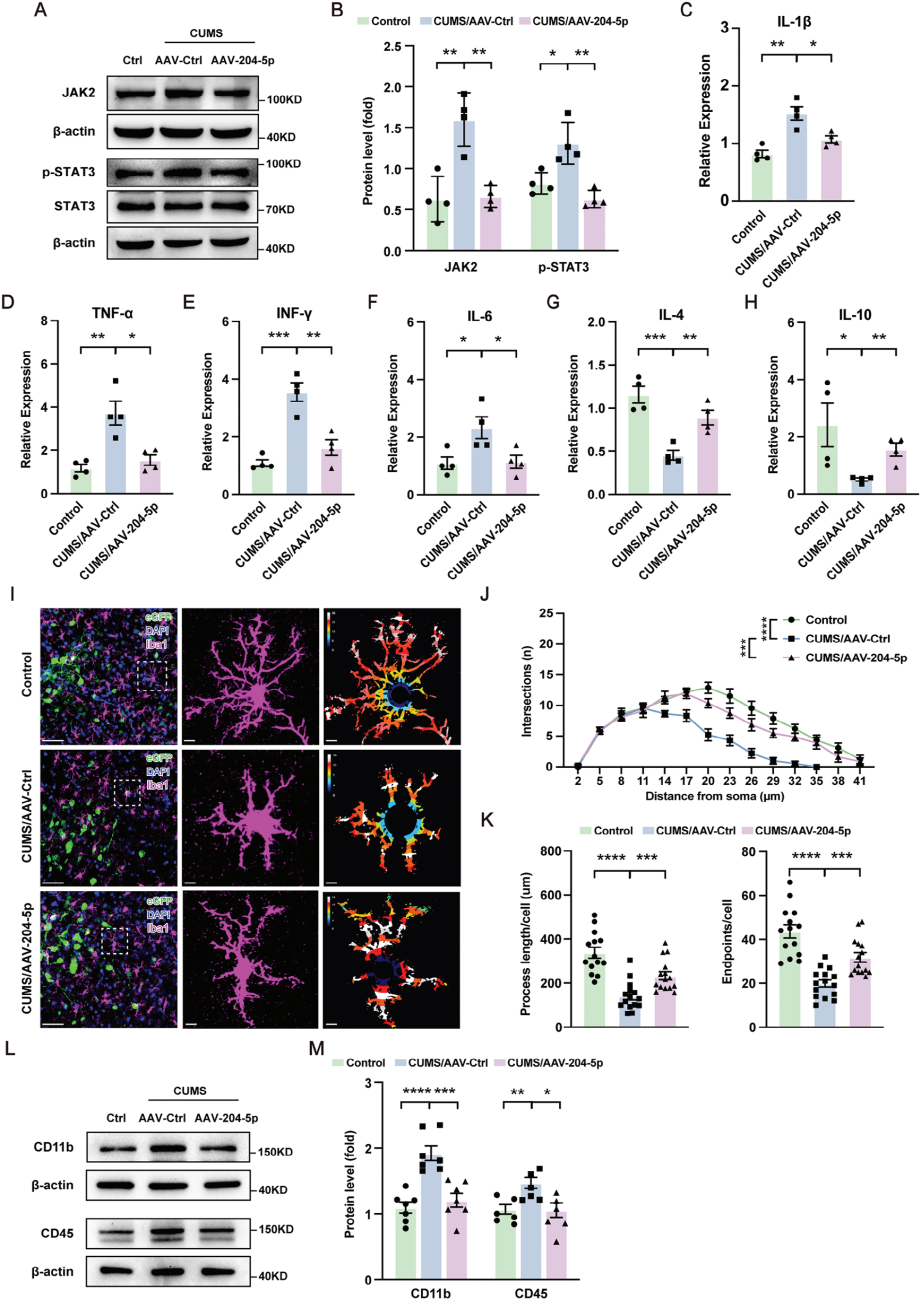
Overexpression of miR‐204‐5p alleviated inflammatory responses within the vmPFC region in rats. A,B) Overexpression of miR‐204‐5p within the vmPFC region decreased protein levels of JAK2 and p‐STAT3 in CUMS/AAV‐204‐5p rats (*n* = 4 rats per group). C–F) Overexpression of miR‐204‐5p within the vmPFC region decreased the expression of pro‐inflammatory cytokines in CUMS/AAV‐204‐5p rats. G,H) Over‐expression of miR‐204‐5p within the vmPFC region increased the expression of anti‐inflammatory cytokines in CUMS/AAV‐204‐5p rats (*n* = 4 rats per group). I) Coronal vmPFC sections stained with DAPI (blue) for nuclei, eGFP (green) for pyramidal neurons, and Iba1 (magenta) for microglia. Representative images of microglia and heat maps of Sholl analysis are shown. Original magnification × 20; scale bar, 40 µm. Zoom‐in images with a scale bar equal to 15 µm. Heat maps with red indicate the radius with the highest number of intersections. J) Sholl analysis showing the number of intersections at different distances from the microglial soma in the indicated groups. K) Left panel showing that an overexpression of miR‐204‐5p significantly increased process length of microglia within the vmPFC region in CUMS/AAV‐204‐5p rats; Right panel showing that an overexpression of miR‐204‐5p significantly increased endpoints of microglia in CUMS/AAV‐204‐5p rats (*n* = 4–5 rats per group). L,M) Expressions of CD11b and CD45 were significantly decreased after overexpression of miR‐204‐5p in CUMS/AAV‐204‐5p rats (*n* = 4–7 rats per group). *p < 0.05, **p < 0.01, ***p < 0.001, ****p < 0.0001, by an unpaired two‐tailed Student's *t*‐test C–H, K, M) and a two‐way ANOVA B, J), data represent means ± SEMs.

**Figure 8:**
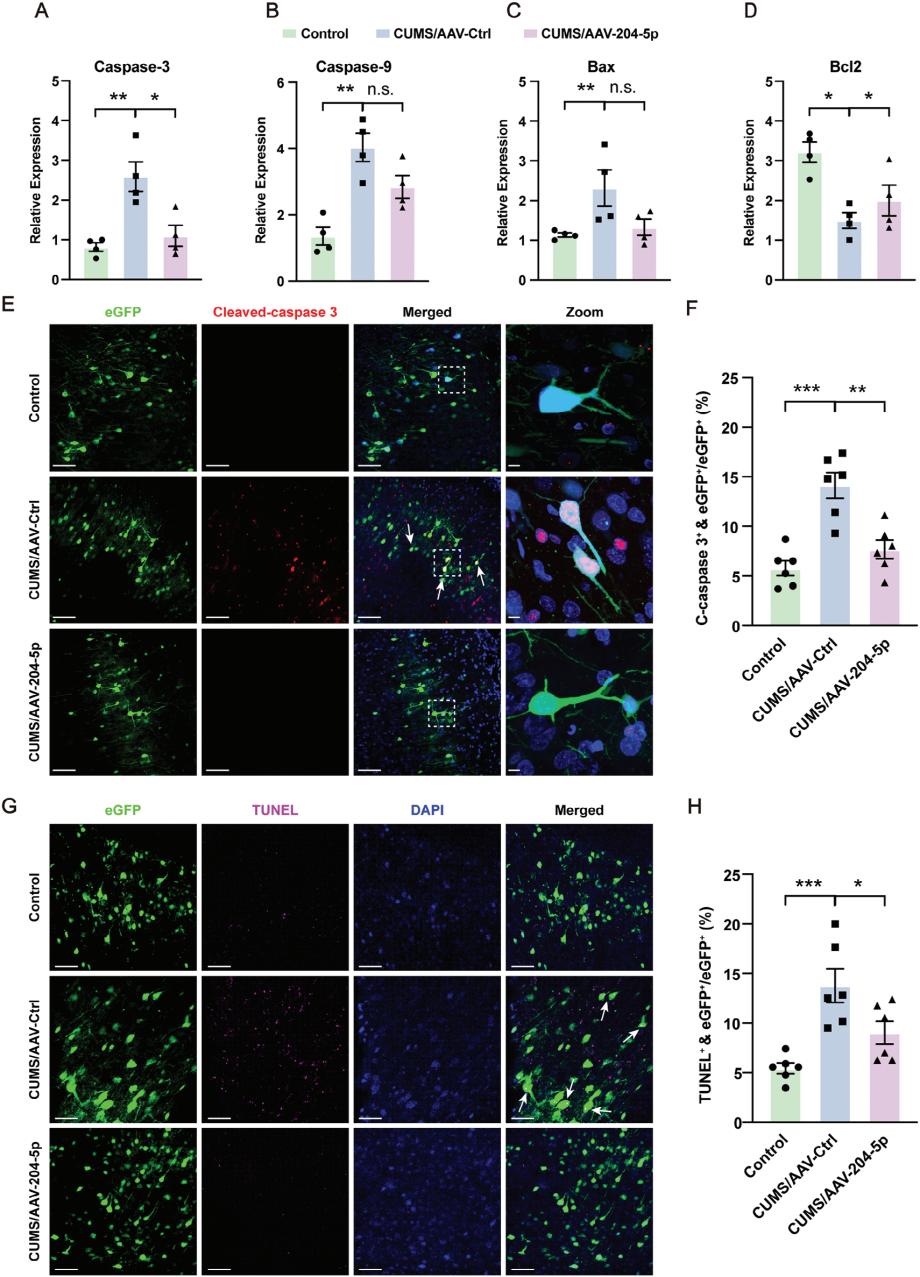
Overexpression of miR‐204‐5p within the vmPFC region rescued neuronal apoptosis and synaptic dysfunction in rats. A–C) Overexpression of miR‐204‐5p decreased the pro‐apoptotic cytokines in CUMS/AAV‐204‐5p rats. D) Anti‐apoptotic cytokines were increased after overexpression of miR‐204‐5p in CUMS/AAV‐204‐5p rats (*n* = 4 rats per group). E) Immunofluorescent staining showing the number of Cleaved‐caspase 3 positive cells (red) and eGFP colocalization (White arrows). Scale bar, 40 µm. Zoom‐in images with a scale bar equal to 15 µm. F) Quantification of the number of Cleaved‐caspase 3 positive cells (red) and eGFP colocalization (*n* = 6 rats per group). G) TUNEL staining. H) Quantification of the number of apoptotic neurons (magenta) and eGFP colocalization (*n* = 6 rats per group). Scale bar, 40 µm. n.s., not significant, *p* > 0.05, *p < 0.05, **p < 0.01, ***p < 0.001, by an unpaired two‐tailed Student's *t*‐test, data represent means ± SEMs.

To investigate whether the rescue of depressive‐ and anxiety‐like behaviors in CUMS rats as achieved with overexpression of 204–5p involves downregulation of the JAK2‐STAT3 signaling pathway, we examined the protein levels of JAK2 and p‐STAT3 in CUMS rats with an overexpression of miR‐204‐5p (**Figure** **7**A,B). The mRNA levels of pro‐inflammatory cytokines which were elevated by CUMS exposure were significantly suppressed by miR‐204‐5p overexpression (Figure 7C–F), while the mRNA expression levels of anti‐inflammatory cytokines were significantly increased (Figure 7G,H). In addition, we observed that this overexpression of miR‐204‐5p restored the CUMS‐induced reductions in intersections along with the shorter processes and endpoints of microglia within the vmPFC region (Figure 7I,K), as well as decreases in protein expressions of CD11b and CD45 (Figure 7L,M). These results revealed that an overexpression of miR‐204‐5p exerted anti‐inflammatory and neuroprotective effects by alleviating microglia‐mediated neuroinflammation injury through inhibiting the JAK2‐STAT3 signaling pathway. Furthermore, we found that upregulation of miR‐204‐5p can block the JAK2‐STAT3 signaling pathway in CUMS rats to then significantly reverse the expression levels of pro‐apoptotic cytokines and anti‐apoptotic proteins (**Figure** **8**A–D), as well as reduce the number of Cleaved‐caspase 3 positive cells, which are increased by CUMS exposure (Figure 8E,F). This upregulation of miR‐204‐5p also significantly restored the nuclear damage and cell apoptosis that occurs in CUMS rats (Figure 8G,H; Figure S5B, Supporting Information). Taken together, these results suggest that miR‐204‐5p may mediate neuroinflammation and apoptosis by targeting the JAK2/STAT3 signaling pathway.

### 2.8 MiR‐204‐5p Regulates Neuronal Synaptic Plasticity within the vmPFC Region in Rats

**Figure 9:**
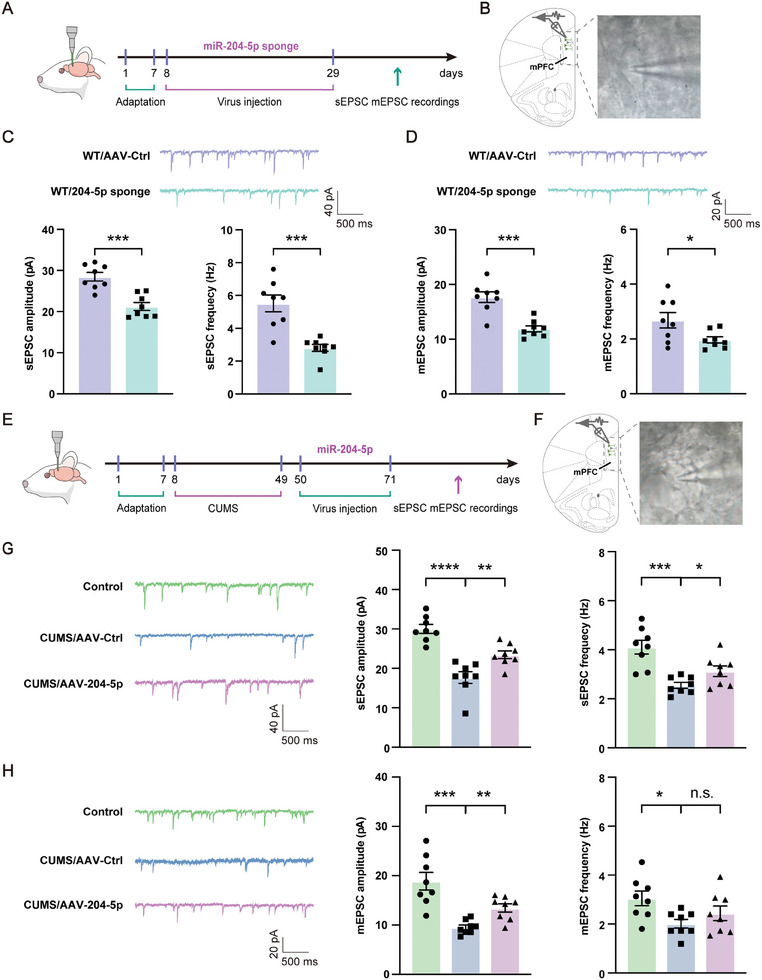
MiR‐204‐5p regulated neuronal synaptic plasticity within the vmPFC region in rats. A) Experimental design. B) Examples of patch‐clamp recordings of vmPFC AAV‐labeled pyramidal neurons under whole‐cell configurations. C) Representative traces and quantitative analysis of sEPSC in vmPFC pyramidal neurons. Knockdown of miR‐204‐5p decreased the amplitudes and frequencies of sEPSC. D) Representative traces and quantitative analysis of mEPSC in vmPFC pyramidal neurons. Knockdown of miR‐204‐5p decreased the amplitudes and frequencies of mEPSC. E) Experimental design. F) Examples of patch‐clamp recordings of vmPFC AAV‐labeled pyramidal neurons under whole‐cell configurations. G) Representative traces and quantitative analysis of sEPSC in vmPFC pyramidal neurons. Overexpression of miR‐204‐5p increased the amplitudes and frequencies of sEPSC in CUMS/AAV‐204‐5p rats. H) Representative traces and quantitative analysis of mEPSC in vmPFC pyramidal neurons. Overexpression of miR‐204‐5p increased the amplitudes and frequencies of mEPSC in CUMS/AAV‐204‐5p rats. (*n* = 8 rats per group), Scale bars, 500 ms, 40 pA. n.s., not significant, *p* > 0.05, *p < 0.05, **p < 0.01, ***p < 0.001, ****p < 0.0001, by an unpaired two‐tailed Student's *t*‐test, data represent means ± SEMs.

To examine whether the knockdown of miR‐204‐5p resulted in synaptic dysfunction like that obtained in CUMS rats, we conducted whole‐cell patch‐clamp recordings in pyramidal neurons from vmPFC coronal slices. A schematic illustrating the experimental design and examples of patch‐clamp recordings of vmPFC AAV‐labeled pyramidal neurons under whole‐cell configuration is contained in **Figure** **9**A,B. Our results showed that knockdown of miR‐204‐5p decreased the amplitudes and frequencies of sEPSC (Figure 9C) and miniature excitatory postsynaptic current (mEPSC) (Figure 9D) of pyramidal neurons in the vmPFC region. In contrast, overexpression of miR‐204‐5p rescued these decreases in amplitudes and frequencies of sEPSC and mEPSC resulting from CUMS exposure (Figure 9E–H). These results provide further evidence that a deficiency of miR‐204‐5p within the vmPFC impaired synaptic transmission. Such an effect may then contribute to the depression/anxiety behavioral responses observed in CUMS rats, whereas overexpression of miR‐204‐5p could alleviate the display of such behaviors in these CUMS rats.

### 2.9 Inhibition of the JAK2/STAT3 Pathway Alleviates Depression/Anxiety Behavioral Displays Induced by a miR‑204‐5p Deficiency

**Figure 10:**
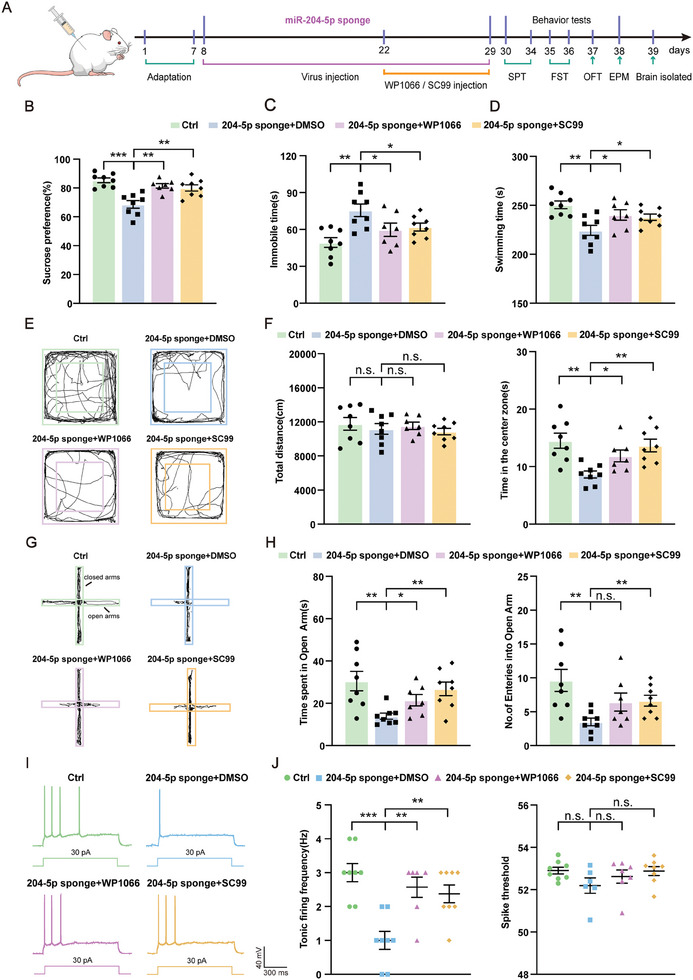
Inhibition of the JAK2/STAT3 pathway alleviated depression‐ and anxiety‐like behaviors as induced by a miR‑204‐5p deficiency. A) Experimental design. B) As compared with that observed in miR‐204‐5p sponge + DMSO rats, WP1066 or SC99 treatment increased sucrose preference scores. C,D) Results of FST. WP1066 or SC99 treatment decreased immobility times and increased swimming times. E,F) Results of OFT. E) Raw traces of locomotor activity in the OFT; F) total distance traveled in the OFT was not significantly different between the three groups (left). As compared with that observed in miR‐204‐5p sponge + DMSO rats, WP1066 or SC99 treatment increased the time spent exploring the center area (right). G,H) Results of EPM. G) Raw traces of movements in the EPM; H) as compared with that observed in miR‐204‐5p sponge + DMSO rats, WP1066 or SC99 treatment increased the number of open arm entries (left) and increased the time spent in the open arms (right). I) Representative traces of action potentials in vmPFC pyramidal neurons. J) WP1066 or SC99 treatment increased tonic firing frequencies (left) but did not change spike thresholds (right) as compared with miR‐204‐5p sponge + DMSO rats. (*n* = 7–8 rats per group). Scale bars, 300 ms, 40 mV. n.s., not significant, *p* > 0.05, *p < 0.05, **p < 0.01, ***p < 0.001, by unpaired two‐tailed Student's *t*‐test, data represent means ± SEMs.

We used, WP1066 or SC99, two inhibitors of the JAK2/STAT3 pathway capable of crossing the blood‐brain barrier and thus achieving therapeutic effects within the central nervous system (CNS),^[^ ^24^ ^]^ to evaluate the contribution of this pathway to the display of depression (**Figure** **10**A). Our behavioral results showed that rats treated with the inhibitor SC99 or WP1066 for 7 days showed a significant decrease in depressive‐ and anxiety‐like behaviors as induced by a miR‑204‐5p deficiency. Specifically, in response to this inhibitor treatment there was a significant increase in the percent of sucrose consumption in the SPT (Figure 10B), along with a decrease in immobility times and increased swimming times in the FST (Figure 10C,D). Treatment with these inhibitors also reversed the anxiety‐like behaviors as observed in the OFT and EPM tests (Figure 10E–H). Moreover, results from whole‐cell patch‐clamp recordings demonstrated that these inhibitors treatment improved tonic firing frequencies, but not spike thresholds of pyramidal neurons within the vmPFC region (Figure 10I,J). These findings provide further support indicating that miR‐204‐5p may directly target the JAK2/STAT3 signaling pathway to induce depression/anxiety behaviors, while inhibition of this pathway ameliorates behavioral disorders in miR‑204‐5p deficient rats. Moreover, they suggest new pathways and approaches that offer the potential for the development of novel interventions in the treatment of depression.

## 3 Discussion

MDD is one of the most common disabling psychiatric disorders and is characterized by alterations in multiple key functions, including mood, cognition, appetite, sleep, and psychomotor activity. The high rates of morbidity and mortality associated with MDD make it one of the most frequent bases for disability and thus exerts a severe socioeconomic burden worldwide.^[^ ^25^ ^]^ Developmentally, anxiety disorders are almost always the primary condition involved with MDD, with onset usually occurring in childhood or adolescence.^[^ ^26^ ^]^ The comorbidity of anxiety and depression is most likely due to the shared genetic vulnerability of both disorders or the possibility for one disorder being an epiphenomenon of the other.^[^ ^27^ ^]^ As chronic stress seems to be a notable risk factor for MDD, identifying the molecular and cellular factors underlying behavioral sensitivity or resilience to stress‐induced behavioral responses represents a crucial issue in elucidating the mechanisms of MDD. Here, we provide evidence that miR‐204‐5p within the vmPFC exerts a key role in the feedback of the stress responses. In specific, our findings represent the first detailed demonstration regarding the function of miR‐204‐5p in regulating the JAK2/STAT3 signaling pathway within the vmPFC brain area, mechanisms that are closely related to the display of the depression and anxiety as observed in the CUMS rat model of depression.

Among the various noncoding RNAs, it has been reported that changes in miRNA expression in CUMS rats are related to the pathogenesis of depression. For example, downregulation of miR‐9‐5p affects dendritic size and spine density of hippocampal neurons, an effect which can be reversed following treatment with the antidepressant, ketamine.^[^ ^28^ ^]^ Work within our group directed at investigating the molecular mechanisms of depression has revealed that miR‐204‐5p may alleviate oxidative stress injury and neuroinflammation responses within the DG hippocampal region of CUMS rats by targeting the downstream RGS12 gene, thus alleviating the depression‐like behavior observed in this rat model of depression.^[^ ^17^ ^]^ It has been well established that the vmPFC represents one of the critical brain regions involved in the pathogenesis of depressive symptoms as revealed by the crucial role it plays in the affective and cognitive deficits of depression.^[^ ^29^ ^]^ Therefore, in this report, we concentrated on changes in miR‐204‐5p within the vmPFC region. As noncoding short‐chain RNAs, miRNAs can suppress the expression of target genes by specifically binding to the 3′‐UTR of mRNA, thus altering biological functions. Therefore, to investigate the downstream mechanism of miR‐204‐5p, we first utilized an online prediction website (TargetScan and MiRNA base) to screen for potential genes. Combining conservatism and free energy of binding sites, JAK2 emerged as a potential target gene. In addition, results from the PPI network analysis, molecular docking, and CO‐IP assays showed that a direct interaction was present between JAK2 and STAT3 proteins.

The JAK/STAT pathway is a newly identified signal transduction pathway shared by multifarious cytokines and can respond to almost all extracellular regulatory signals. It involves tyrosine kinase‐associated receptors and two protein families, JAKs and STATs. Currently, four types of JAK (JAK1, JAK2, JAK3 and TYK2) and six of STATs (STAT1, STAT2, STAT3, STAT4, STAT5A, STAT5B, and STAT6) have been identified.^[^ ^30^ ^]^ Among them, JAK2 and STAT3 are regarded as the oldest and most conserved isomers and the most prominent isomers that have been shown to broadly affect the adaptability of cells to environmental stimulation or stress. The JAK2/STAT3 signaling pathway can mediate apoptosis‐related gene expression and the upregulation of antiapoptotic genes which are beneficial in reducing neuronal apoptosis.^[^ ^31^ ^]^ In our study, results from GO enrichment analysis of the downstream targets indicated that miR‐204‐5p was most likely to be involved in the pathogenesis of depression by activating transcription factors and mediating neuroinflammation and apoptosis. And western blotting assay results revealed that protein expression levels of the JAK2/STAT3 signaling pathway were significantly activated after knockdown of miR‐204‐5p in rats.

Neuroinflammation contributes to depression through promoting neurotransmitter dysregulation, damaging neurons, altering neuronal activity in specific depression‐related brain regions, and producing a HPA axis dysfunction.^[^ ^32^ ^]^ Our results demonstrated that a miR‐204‐5p deficiency within the vmPFC region induced anxiety‐ and depression‐like behaviors in rats, effects which were accompanied by increased expressions of the pro‐inflammatory cytokines, IL‐1β, TNF‐α, IFN‐γ and IL‐6, as well as reduced expressions of the anti‐inflammatory cytokines, IL‐4 and IL‐10 within the vmPFC region. In addition, a miR‐204‐5p deficiency also augmented glia activation and protein expressions of CD11b and CD45. Apoptosis is a critical neuronal process involved with the progression of neuronal injury and consequent depression‐like behavior. In this study, we utilized qPCR, TUNEL, and Western blotting assays to evaluate the effects of miR‐204‐5p on cell apoptosis. We also identified the cleaved‐caspase 3 protein as providing a major indicator for cell apoptosis. With the knockdown of miR‐204‐5p in the vmPFC there were significant increases in the pro‐apoptotic cytokines, Caspase‐3, Caspase‐9, and Bax, and decreases in the anti‐apoptotic protein, Bcl2. Notably, these changes in expression levels were all reversed when miR‐204‐5p was overexpressed in CUMS rats. Moreover, results from our immunofluorescent assay showed that the levels of Cleaved‐caspase 3 positive cells and TUNEL‐positive cells were significantly increased in miR‐204‐5p deficient rats, suggesting that miR‐204‐5p may mediate neuroinflammation and apoptosis by directly targeting the JAK2/STAT3 signaling pathway.

As an approach to more specifically determine whether the JAK2/STAT3 signaling pathway may contribute to the enhancement of neuroinflammation and apoptosis, thus producing the dysregulation of neuroplasticity via a miR‐204‐5p deficiency, we overexpressed miR‐204‐5p within the vmPFC region in CUMS rats to downregulate the JAK2/STAT3 signaling pathway. This miR‐204‐5p overexpression significantly ameliorated the depression‐ and anxiety‐like symptoms in these CUMS rats, effects which were accompanied decreases in inflammatory responses and apoptosis. Simultaneously, this miR‐204‐5p upregulation also significantly reduced the high expression levels of pro‐inflammatory cytokines and increased anti‐inflammatory cytokines levels. Moreover, from our whole‐cell patch‐clamp recordings, we found that miR‐204‐5p overexpression reversed the decreases in frequency and amplitude of miniature and spontaneous EPSCs, which were induced by CUMS exposure. As a means to assess whether the JAK2/STAT3 signaling pathway may contribute to depressive‐ and anxiety‐like behaviors in miR‐204‐5p deficient rats, we injected SC99 or WP1066, the JAK2/STAT3 signaling pathway inhibitor, for 7 days after virus infection 2 weeks in miR‐204‐5p knockdown rats. With these inhibitors treatment, depression‐ and anxiety‐like symptoms were significantly decreased, while tonic firing frequencies of pyramidal neurons in the vmPFC region were increased. Taken together, these results suggest that the JAK2/STAT3 signaling pathway may act as a downstream target in response to a miR‐204‐5p deficiency, thereby contributing to the neuronal injury and behavioral disorders observed in these rats.

It is important to note that there remain a number of issues to be resolved before an effective use of miRNA can be employed in the treatment of MDD. Currently, research on miRNAs mainly focuses on the relationship between a single miRNA and its target gene, with relatively little work directed to studies on the co‐regulatory networks of miRNAs. Another major challenge is to identify a carrier drug that would enable miRNA transport through the blood−brain barrier as well as to certain tissues. In this study, we used AAV as a carrier to deliver miR‐204‐5p with high transfection efficiency, however, this would not represent a readily transferrable approach for use in clinical practice, which will then require a large number of trails prior to its implementation. Research involved with the use of exosomes has demonstrated a more feasible/practical approach for the delivery of miRNA and, in fact, in recent years, miRNA therapy has been used in the treatment of tumors^[^ ^33^ ^]^ and hepatitis C virus (HCV).^[^ ^34^ ^]^ Accordingly, the problems associated with the use of miRNA therapy will likely be gradually resolved to bring new hope for patients with MDD.

In conclusion, these findings reveal some new insights into the possible molecular mechanisms for the neuroprotective effects of miR‐204‐5p. In specific, we show that a miR‐204‐5p deficiency contributes to the enhancement of neuroinflammation and apoptosis involved with the pathogenesis of behavioral disorders via activating its downstream target, the JAK2/STAT3 signaling pathway, which then promotes neuronal deterioration in rats. Taken together, these findings suggest that targeting mechanisms involved with the inhibition of neuroinflammation and apoptosis may serve as a new therapeutic strategy for the treatment of stress‐related neurological disorders.

## 4 Experimental Section

### Animals and Housing Conditions

Male Wistar rats (120–140 g, ≈5 weeks old) were purchased from the Experimental Animal Centre of Shandong University. Animals were maintained under standard laboratory conditions at 22 ± 2 °C, a humidity of 35–40%, with a 12 h light/dark cycle and free access to a standard diet and drinking water. Rats were acclimatized to this environment for one week prior to the start of the experiment. All procedures were approved by the Animal Care and Use Committee of Shandong University (ECSBMSSDU‐2022‐2‐65) and were performed in accordance with the National Institutes of Health Guide for the Care and Use of Laboratory Animals.

### CUMS Model

Following their one‐week acclimatization period, the rats were subjected to the CUMS regime for 6 weeks. During this regime, the experimental animals received 1–2 types of random, continuous, non‐repetitive daily stimulation consisting of: 12 h fasting and water deprivation, Day/night reversal (24 h of continuous light or darkness), Tilting of the cage (45° for 12 h), Wet cage (wet bedding for 24 h), Tail clamping (the tail of the animal was clamped with hemostatic forceps for 5 min), Forced swimming in ice water for 5 min and physical restraint for 1 h. None of the same stressors were applied in succession.

### Drug Treatments—WP1066

WP1066 (S2796), which is an inhibitor of STAT3 phosphorylation, is produced by Selleck Chemicals (Houston, Texas, USA). Named for Waldemer Priebe, WP1066, was developed from a series of small molecular STAT3 inhibitors designed from the caffeic acid benzyl ester/AG490 scaffold^[^ ^35^ ^]^ as an agent which can optimally inhibit JAK2/STAT3 interactions and subsequent phosphorylation of STAT3 at tyrosine 705.^[^ ^36^ ^]^ The dose and route of WP1066 administration were based upon previous studies with minor modifications^[^ ^37^ ^]^ and consisted of a 30 mg kg^−1^ intraperitoneal injection for 7 days, with DMSO (0.1%) serving as the vehicle control.

### Drug Treatments—SC99

SC99 (S1349), which is a novel specific inhibitor of JAK2/STAT3 activation, was produced by Selleck Chemicals (Houston, Texas, USA). SC99 exerts no effects on other transcription factors such as nuclear factor‐k‐gene binding (NF‐κB) or protein kinases such as protein kinase B (PKB) and extracellular signal‐regulated kinase (ERK), which were associated with the STAT3 signaling pathway.^[^ ^38^ ^]^ The dose and route of SC99 administration is based upon a previous study with minor modifications and consisted of an intracerebroventricular injection (i.c.v.) for 7 days at 15 mmol L^−1^ in 15 uL with DMSO (0.1%) serving as the vehicle control.

### Cell Culture

Primary cultures of mixed glial cells and primary neurons were prepared from newborn P1 rat pups as described previously.^[^ ^39^ ^]^ Purity of the microglia, astroglia, and neuron preparations were verified by staining with anti‐Iba1 (1:1000, Wako Chemicals, 019–19741), anti‐GFAP (1:200, Proteintech, 16825‐1‐AP) and anti‐MAP2 (1:200, Cell Signaling Technology, 4542S), respectively. BV2 cells were purchased from Iennio (Guangzhou, China) and cultured in DMEM supplemented with 10% FBS and 1% penicillin/streptomycin (Thermo Fisher Scientific) at 37 °C in 5% CO2.

The cells were transferred to serum‐free medium, exposed to LPS (100 ng mL^−1^) for 24 h, collected, and then assayed for determination of miR‐204‐5p expression levels.

### Behavioral Tests

All behavioral tests were performed during the light phase (1100–1700 h). Behavioral analyses were performed by an investigator blinded as to the experimental condition.

### Behavioral Test—Sucrose Preference Test (SPT)

The SPT is one of the most effective methods to detect anhedonia in the CUMS rat model of depression. This test was performed after 6 weeks of CUMS and the specific experimental procedures were as follows. Animals were single‐housed and habituated with two bottles of 1% sucrose solution for 24 h, followed by one bottle of 1% sucrose solution and one bottle of tap water for 24 h. Following a 24 h period of fasting and water deprivation, rats were exposed to one bottle of 1% sucrose solution and one bottle of water for 2 h in the dark phase. After 1 h, the position of two bottles was changed to eliminate any potential confounding related to position preference. During the test, the general procedures and placements of the bottles were consistent to reduce the influence of human factors on the experimental results. Sucrose preference = sucrose consumption/(sucrose consumption + water consumption) ×100%.

### Behavioral Test—Forced Swimming Test (FST)

Animals were individually placed in a cylinder (diameter = 40 cm, height = 80 cm) containing water (23–25 °C) for a 15 min forced swimming training session. Water depth was set to prevent animals from touching the bottom with tails or hind limbs. Twenty‐four hours later, each rat was again placed in the cylinder for a 5 min test session, during which immobility and swimming times were recorded by an observer blinded as to the treatment condition of the animal. Immobility was defined as the amount of time the animals remained floating or motionless with only movements necessary for maintaining a buoyant position in the water. The cylinder was cleaned and deodorized before each test and the ambient environment was quiet with a consistent illumination during the test to eliminate any potential interference of environmental and/or human factors.

### Behavioral Test—Open Field Test (OFT)

The OFT was used to analyze spontaneous exploratory activity and evaluate the level of anxiety. Rats were placed in the center of an arena (100 cm × 100 cm × 50 cm) with a dim light illumination for a 5 min period. Spontaneous locomotor activity was recorded using a camera. Total distance traveled (cm) and time spent in the center area during the 5‐minute period were automatically recorded using tracking software (SMART 2.5, Panlab).

### Behavioral Test—Elevated‐Plus Maze Test (EPM)

The maze consisted of a plus‐shaped apparatus, with two “open” arms (60 cm × 12 cm, without walls) and two “closed” arms (60 cm × 12 cm, with walls extending 30 cm in height). Each rat was placed in the central area of the maze facing an open arm and allowed to explore for 5 min. The room was kept quiet during the entire test and the apparatus was cleaned with 75% ethanol after each trial. The total distance traveled in the four arms was recorded using tracking software (SMART 2.5, Panlab). The time spent and number of entries into the open arms were recorded.

### Stereotaxic Injection of the AAV Virus

The viruses used in this study to knockdown or overexpress miR‐204‐5p were constructed from GENEchem (Shanghai, China). After completion of all pre‐treatment behavioral tests, a subgroup of rats from the control and CUMS groups were randomly selected for virus infusion. Rats were deeply anesthetized with sodium pentobarbital (50 mg kg^−1^) and placed in a stereotaxic frame (Stoelting, USA) for a bilateral infusion of 1.2 µL (0.12 uL min^−1^) of purified and concentrated AAV9 virus into the vmPFC (AP: +3.24 mm; ML: ±0.5 mm; DV: −4.8 mm). After the injection was completed, the infusion cannula remained at the site for an additional 5 min before being slowly and completely withdrawn. After surgery, animals were allowed to recover from anesthesia while under a heating pad. The AAV9‐rno‐miR‐204‐5p virus (primer sequence: UUCCCUUUGUCAUCCUAUGCCU) was constructed to overexpress miR‐204‐5p while the AAV9‐rno‐miR‐204‐5p‐sponge virus (inverse complementary sequence: AGGCATAGGATGACAAAGGGAA) was used to block miR‐204‐5p. Post‐treatment behavioral tests or biochemical assays were performed at 3 weeks after injection, and injection sites were inspected to verify an accurate placement after all behavioral tests were completed. Only rats with correct injection sites were used for analyses in the subsequent assays.

### Brain Dissection and Slice Preparation

Twenty‐four hours after behavioral tests, rats from each group were anesthetized with sodium pentobarbital (50 mg kg^−1^) and transcardially perfused with 0.9% NaCl followed by 4% paraformaldehyde (PFA). Brains were removed and post‐fixed in 4% PFA overnight at 4 °C followed by a graded dehydration. Brain samples were cut into serial coronal frozen sections, with prefrontal cortex sections then being selected for immunofluorescent staining.

### Immunofluorescent Assay

Tissue samples were frozen in Optimal Cutting Temperature compound (OCT, TissueTek) with sections (40 µm) then cut on a freezing microtome (Leica), collected in PBS, and stored at −20 °C for further use. Sections were permeabilized with PBST (0.1 m PBS with 0.3% Triton X‐100) for 30 min and blocked with PBST containing 5% normal goat serum for 1 h at room temperature to block nonspecific staining. Then, sections were incubated with the primary antibody in PBST with 1% normal goat serum overnight at 4 °C. The following primary antibodies were used in the experiments: anti‐PSD95 (1:50, Santa Cruz, sc‐32290), anti‐vGluT1 (1:5000, abcam, ab227805), anti‐Iba1 (1:1000, WAKO Chemicals, 019–19741) and anti‐Cleaved‐Caspase 3 (1:200, Cell Signaling Technology, 9661S). The sections were washed three times in PBST and incubated with the indicated secondary antibodies for 1.5 h at room temperature on the following day. Secondary antibodies used in the study were: Alexa Fluor 488 (1:1000, Invitrogen, A‐11094) and Alexa Fluor 568 (1:1000, Invitrogen, A‐11011). Sections were then stained for 30 min with 4′,6‐diamidino −2‐phylindole, dihydro‐chloride (DAPI, Beyotime, c1002) diluted 1:1000 in PBS and mounted in antifade medium after three washes with PBST. Finally, sections were imaged using confocal microscopy (LSM880; Zeiss) and processed with use of ZEN software. Fluorescent intensity analyses were performed using Image J (v.7.4.2). For analyses of synapses, imaris software (Bitplane) was used to perform a 3D reconstruction and surface rendering, For microglia, those located at a distance of >20 µm from the plaque surface were included for analysis and quantification.

### TUNEL Assay

Apoptosis of cells was determined with use of the one‐step TUNEL apoptosis assay kit (Vazyme, A113‐01) according to the manufacturer's protocol. Briefly, cells were treated and subsequently fixed with 4% paraformaldehyde at room temperature for 30 min, then incubated with 0.1% Triton X‐100 for 10 min and washed three times in PBS for 5 min each. The cells were then incubated with 50 µl of TUNEL reaction mixture for 1 h at 37 °C in the dark, with nuclei stained using DAPI. Finally, the expression of apoptotic cells was observed with use of confocal microscopy (LSM880; Zeiss) and processed using ZEN software.

### Western Blotting

Rats were anesthetized with an intraperitoneal injection of sodium pentobarbital (50 mg kg^−1^). Bilateral prefrontal cortex tissue samples were isolated and lysed in RIPA buffer containing a cocktail of protease/phosphatase inhibitors. Protein concentrations were determined with use of the BCA protein assay kit (Pierce Bio‐technology, Inc., US), with 10–20 µg of protein in each lane being separated on a 10% SDS–PAGE gel and transferred for western blot analysis. Protein samples were detected with use of the following antibodies: anti‐JAK2 (1:500, Proteintech, 17670‐1‐AP), anti‐p‐STAT3 (1:500, Cell Signaling Technology, 9145S), anti‐STAT3(1:200, Santa Cruz, sc‐8019), anti‐CD11b (1:1000, Abcam, ab133357) and anti‐CD45 (1:1000, Abcam, ab10558). The secondary antibody was a horseradish peroxidase‐conjugated antibody (1:5000, SC‐2030, Santa Cruz). The blots were assessed using an enhanced chemiluminescence detection kit (GE Healthcare, Bucking Hamshire, UK) with all bands being analyzed using Image‐J software (v.7.4.2).

### RNA Preparation and Quantitative Real‐Time PCR

For RNA isolation from brain tissue, the TRIzol method (ACCURATE BIOLOGY, AG21102) was used. Total RNA was quantified using a Nano Drop ND‐1000 spectrophotometer (Nano Drop Thermo, Wilmington, DE) and an equal quantity of RNA (500 ng) was reverse transcribed to synthesize cDNA. Relative amounts of target genes were calculated using the 2^−ΔΔCt^ method with quantitative real‐time polymerase chain reaction (qPCR). For mRNA, qPCR was performed in a total reaction volume of 20 µL containing 10 µl SYBR Green Master mix (Nobelab Biotech, R605, China) for 40 cycles (15 s at 95 °C and 1 min at 60 °C). The mRNA levels of GAPDH were used as the internal control. Expression levels of miRNA were determined using quantitative real‐time quantitative PCR with The All‐in‐OneTM miRNA RT‐PCR Detection Kit (Gene Copoeia, QP010, USA). Real‐time quantitative PCR analysis was performed on a Bio‐Rad Cycler system (BioRad, Hercules, CA) with Rno‐U6 serving as a loading control for the sample to test for miRNA. All samples were repeated four times to reduce the potential for error and all special primers were obtained from BGI Genomics Co., Ltd (Table S1 Supporting Information).

### vmPFC Slice Preparations and Electrophysiological Analysis

Three weeks after AAV injection, rats were anesthetized with pentobarbital sodium (50 mg kg^−1^, i.p.) and immediately decapitated. The brains were transferred to an ice‐cold saturated oxygen cutting recovery solution containing (in mm): 30 glucose, 2.5 KCl, 26 NaHCO_3_, 7 MgSO_4_, 1 NaH_2_PO_4_, 1 CaCl_2_, 119 choline chloride, 1 kynurenic acid, 3 sodium pyruvate, and 1.3 sodium L‐ascorbate. vmPFC slices (300 µm in thickness) were prepared using a vibratome (VT‐1200S, Leica), and all slices were then transferred to a cutting recovery solution. Slices were incubated for a minimum of 60 min at 36 °C in the recovery solution containing (in mm): 85 NaCl, 2.5 KCl, 1.25 NaH_2_PO_4_, 0.5 CaCl_2_, 4 MgCl_2_, 24 NaHCO_3_, 25 glucose and 50 sucrose. Slices were then transferred to a recording chamber at room temperature (20–24 °C) and were continuously perfused with standard artificial cerebrospinal fluid (ACSF) containing (in mm): 127 NaCl, 25 NaHCO_3_, 25 D‐glucose, 2.5 KCl, 1.25 NaH_2_PO_4_, 2 CaCl_2_ and 1 MgCl_2_. Glass electrodes (5–7 MΩ) were used for electrophysiological recordings. For recordings of mEPSC, neurons were clamped at −70 mV in the presence of 1 µm tetrodotoxin and 100 µm picrotoxin in standard ACSF. Data were analyzed using the Mini Analysis Program (Synaptosoft, Decature, GA) with an amplitude threshold of 5 pA for mEPSC and sEPSC analyses.

### Golgi Staining

Golgi staining was performed using the FD Rapid Golgi Stain kit (FD Neuro Technologies, PK401). In brief, freshly dissected brains were immersed in solutions A and B for two weeks at room temperature and then transferred into solution C for 72 h at 4 °C in the dark. Recently obtained brain slices (100 µm) were prepared using a vibratome (Leica, VT 1200S) and sections were mounted on gelatine‐coated slides. After washing, the slides were dehydrated in four gradient concentrations of ethanol, four times for 5 min each time. Finally, images of dendritic spines were acquired using a 100 × objective, with dendritic spine densities and the number of branches being assessed using Image J (v.7.4.2) software.

### Sparse Labeling

Rats were anesthetized using an i.p. injection of pentobarbital sodium (50 mg kg^−1^). The AAVs were bilaterally injected into the vmPFC in a volume of 1 µL containing pAAV‐PTRE‐tight‐NLS‐Cre‐WPRE (H8142, OBiO Technology, Shanghai) and pAAV‐hSyn‐DIO‐WPRE (H6884, OBiO Technology, Shanghai). After 3 weeks, the brains were extracted, fixed in 4% PFA overnight at 4 °C, and dehydrated in 20% and 30% sucrose in PBS buffer. Consecutive 100 µm brain slices were washed 3 times with PBS. Dendritic spines in the vmPFC region were visualized using confocal microscopy (Dragonfly 200, Andor, UK) and assessed using Image J (v.7.4.2) software.

### Molecular Docking

The protein structures of JAK2 and STAT3 derived from the rats were obtained from the UniProt database (https://www.uniprot.org/). This database provides sequences and functional information for proteins as contained within the literature. Gramm‐x server (http://vakser.bioinformatics.ku.edu/resources/gramm/grammx) was used for assessing rigid molecular docking between proteins to evaluate any possibility of their interactions. JAK2 was defined as a receptor while STAT3 as a ligand. Among the output models, the first one obtained was used as the final model and then PyMOL and LigPlot+ were used for visualization.

### Statistical Analysis

GraphPad Prism 8.0.1 was used for statistical analysis, processing, and mapping of the data. *t*‐Tests were used to evaluate statistical significance between two groups and one‐ or two‐way analysis of variance (ANOVA) were used to evaluate statistical significance among three or more groups. Correlation analysis was used one‐way ANOVA with Bonferroni's multiple‐comparison test, *p* values shown in n were determined by Pearson's r. All data in the experiment are expressed in means ± SEM. Statistical significance was indicated as n.s., not significant, *p* > 0.05, *p < 0.05, **p < 0.01, ***p < 0.001 and ****p < 0.0001.

## Conflict of Interest

The authors declare no conflict of interest.

## Author Contributions

S.Y. contributed to study conception and design, and drafted the manuscript, provided funding acquisition; S.C. and X.Z. revised the manuscript and provided funding acquisition; X.C. performed animal perfusion, qPCR analysis, western blot, IF images, electrophysiological recording, analyzed electrophysiological data, prepared figures, and help drafted and revised the manuscript; Y.G. performed qPCR analysis, western blot, GO enrichment analysis; Y.W. performed tissue RNA and protein extraction, constructed animal models and performed behavioral tests; Y.L. performed mice surgeries, viral injection, qPCR analysis; T.L. performed behavioral tests and tissue preparation. All authors read and approved the manuscript prior to submission.

## Supporting information



Supporting Information

Supporting Information

## Data Availability

The data that support the findings of this study are available from the corresponding author upon reasonable request.
