# Supplementary material for: MicroRNA‐204‐5p Deficiency within the vmPFC Region Contributes to Neuroinflammation and Behavioral Disorders via the JAK2/STAT3 Signaling Pathway in Rats
Source: Adv Sci (Weinh). 2025 Jan 10;12(10):2403080. doi: 10.1002/advs.202403080 (PMC11905084; doi:10.1002/advs.202403080)
Supplement: Supplementary file 1 — Supporting Information [file ADVS-12-2403080-s001.docx]

**Supplementary Information**

**MicroRNA-204-5p deficiency within the vmPFC region contributes to neuroinflammation and behavioral disorders via the JAK2/STAT3 signaling pathway in rats**

Xiao Chen^1^, Yeting Gan^1^, Kaiqi Zhang^1^, Yuhan Wu^1^, Ye Li^1^, Tian Lan ^1^, Xianghua Zhuang^2#^, Shihong Chen^2#^, Shuyan Yu^1, 3#^

1. Shandong Key Laboratory of Mental Disorders and Intelligent Control, The Second Hospital of Shandong University, School of Basic Medical Sciences, Shandong University, Jinan, Shandong, 250012, China;
2. Department of Endocrinology and Metabolism, The Second Hospital of Shandong University, Jinan, Shandong, China

3. Department of Medical Psychology and Ethics, School of Basic Medical sciences, Cheeloo College of Medicine, Shandong University, Jinan, Shandong, 250012, China;

^#^ Corresponding author: Shuyan Yu,

E-mail address: shuyanyu@sdu.edu.cn

Tel: +86-0531-88383902; fax: +86-0531-88382502


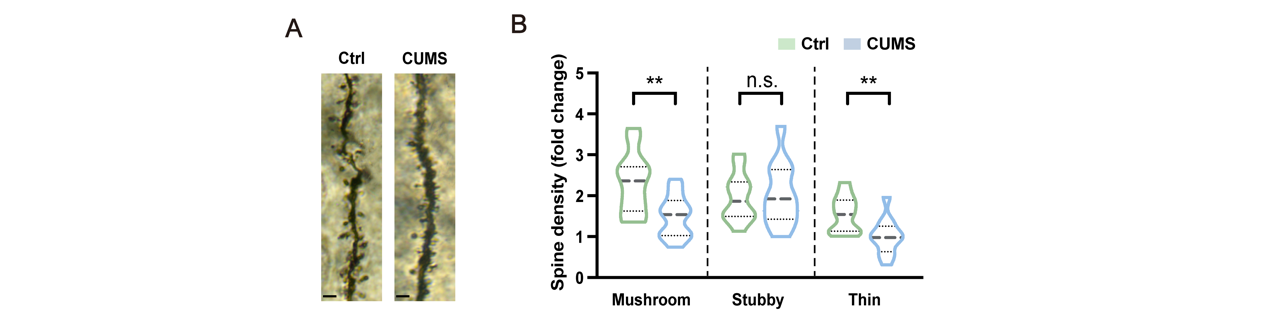


**Figure S1.** CUMS exposure decreased synaptic density and damaged synaptic plasticity within mPFC region in rats. A) Representative dendrites from mPFC region with Golgi staining. Scale bar, 5 µm. B) CUMS decreased the density of three types of dendritic spines (n = 3 to 4 rats per group, 8-10 dendrites per group were counted). n.s., not significant, P > 0.05, **p < 0.01, by a two-way ANOVA, data represent means ± SEMs.


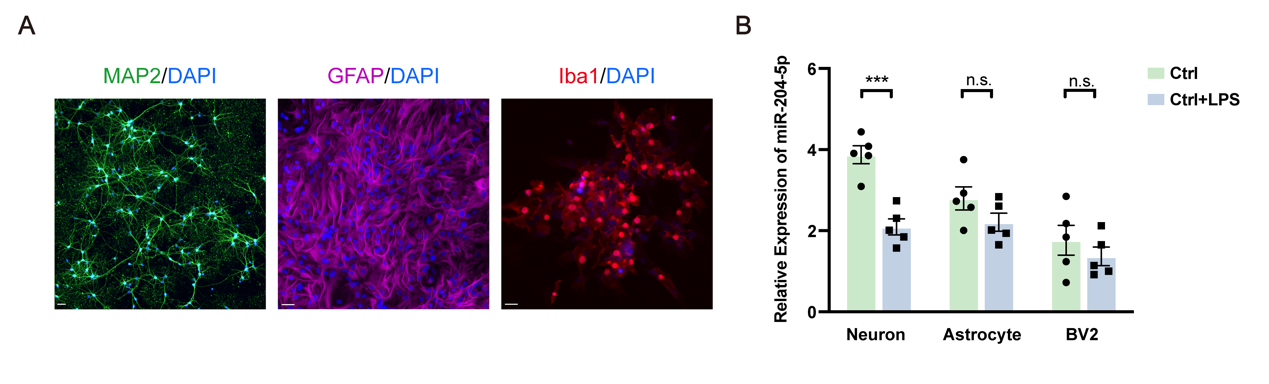


**Figure S2.** miR-204-5p was highly expressed and more sensitive to LPS stimulation in the neuron than that in microglia and astroglia. A) Immunofluorescence staining of MAP2, GFAP and Iba-1 for identifying primary hippocampal neurons, microglia and astroglial revealed a pure culture. B) Quantitative PCR data of miR-204-5p derived from cultured primary neuron, astroglia and BV2 compared with LPS treatment. n = 5 per group. n.s., not significant, P > 0.05, ***p < 0.001, by a two-way ANOVA, data represent means ± SEMs.


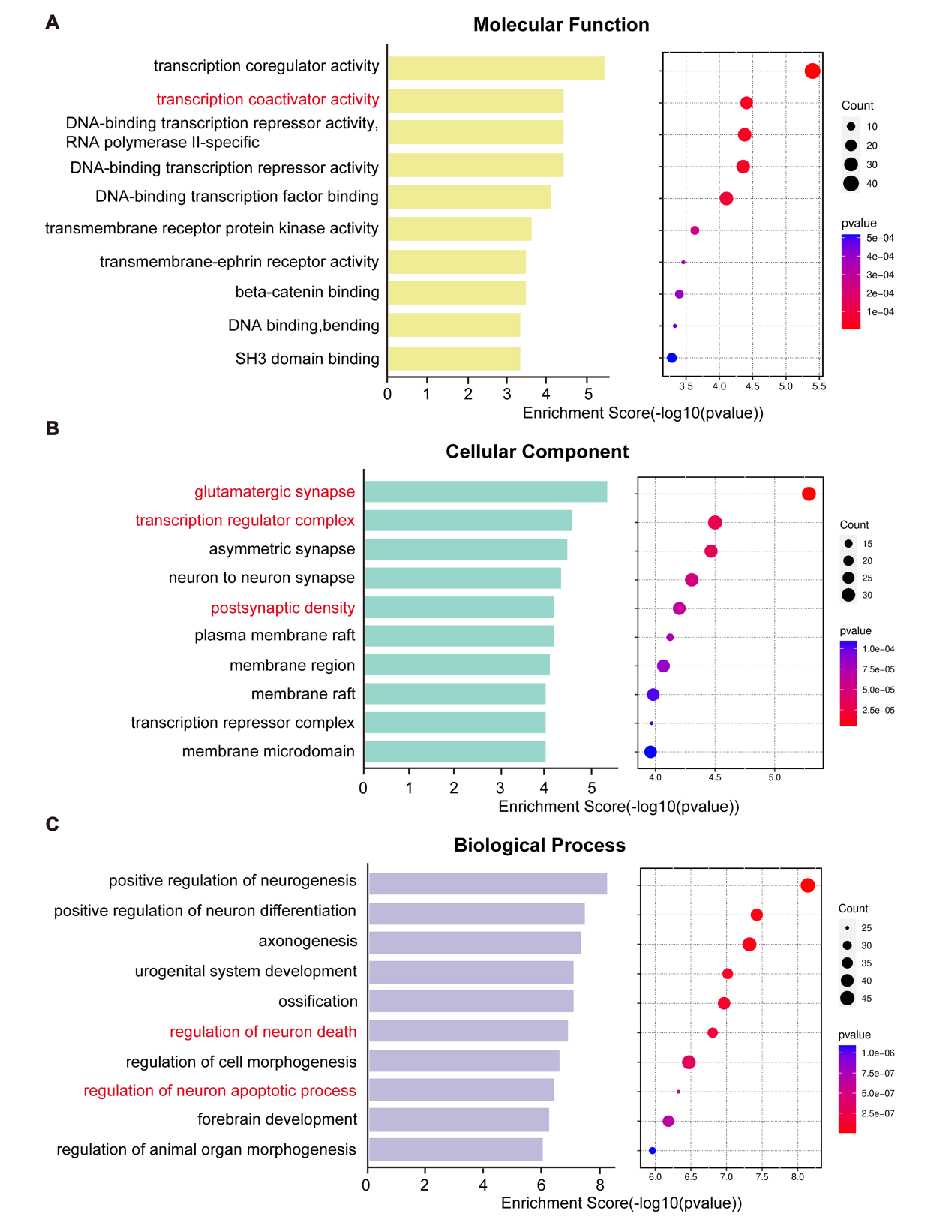


**Figure S3.** MiR-204-5p target gene prediction and GO enrichment analysis. A) GO analysis regarding molecular function. B) GO analysis regarding cellular component. C) GO analysis regarding biological processes.


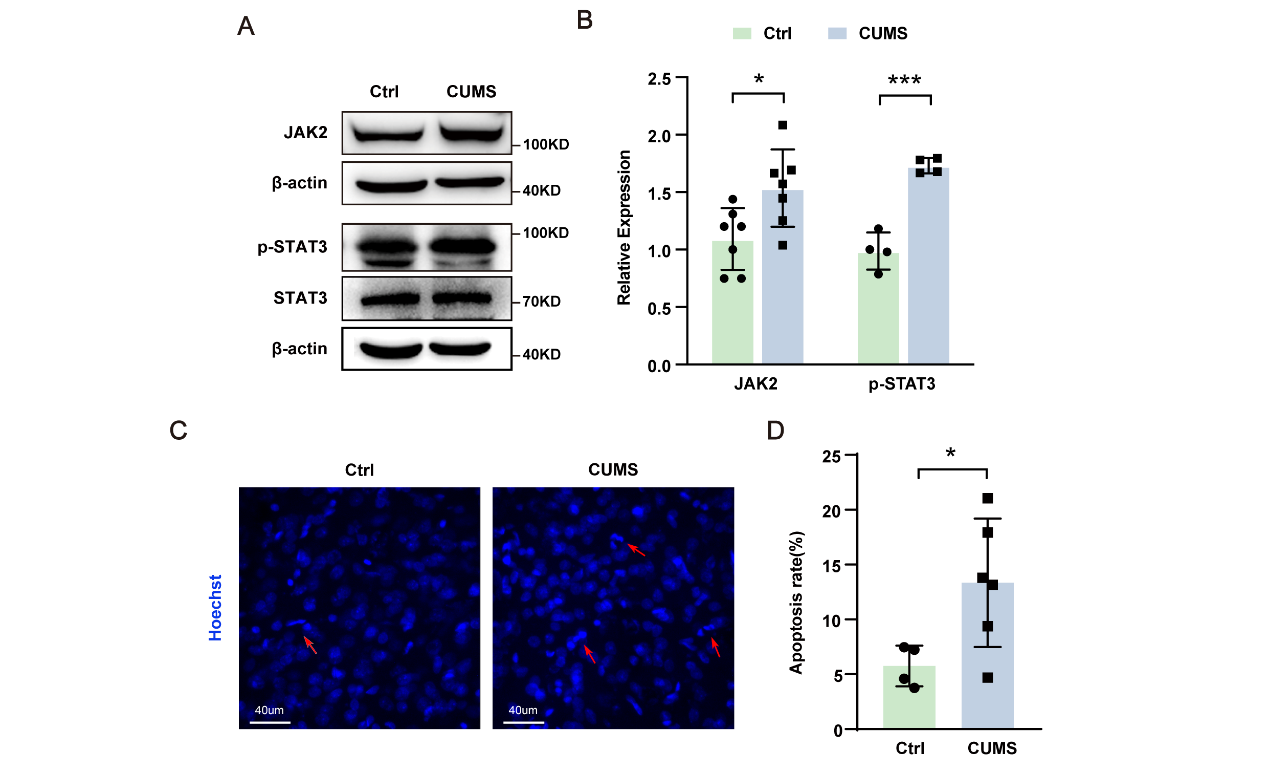


**Figure S4.** CUMS-expose causes neuron apoptosis and the activation of JAK2/STAT3 signaling pathway. A-B) The proteins level of JAK2 and p-STAT3 were increased in CUMS rats (n = 4-7 rats per group). C) Hoechst-33258 staining showed the morphological changes in nuclei. Scale bar is 40 µm. D) The apoptosis rate within mPFC region was significantly increased in CUMS rats (n = 4-6 rats per group). *p < 0.05, ***p < 0.001, by a unpaired two-tailed Student’s t-test (D) and a two-way ANOVA (B), data represent means ± SEMs.


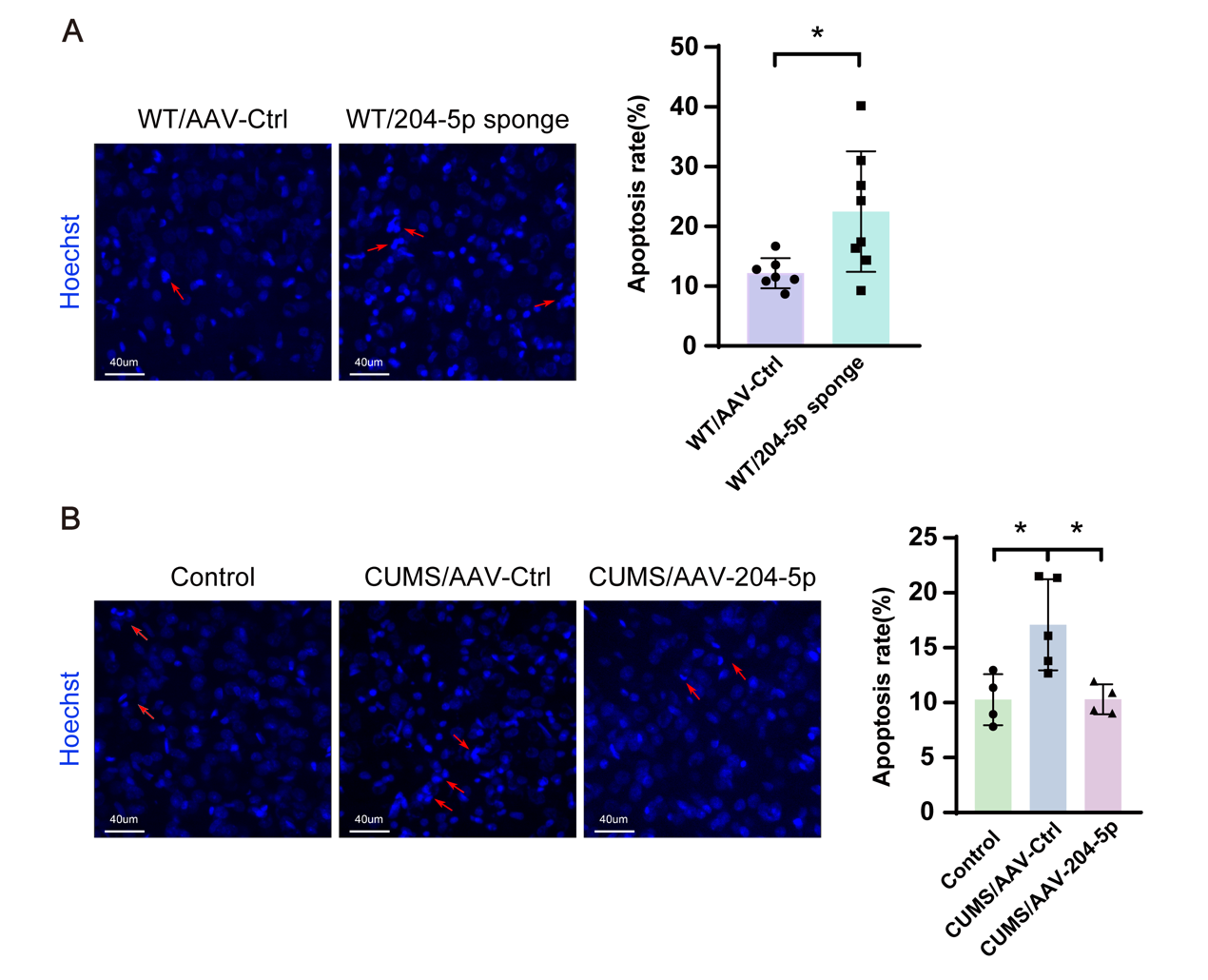


**Figure S5.** A) Hoechst-33258 staining showed the apoptosis rate within mPFC region was significantly increased after knock-down of miR-204-5p. B) Hoechst-33258 staining showed overexpression of miR-204-5p significantly decreased the apoptosis rate within mPFC region compared with CUMS/AAV-Ctrl rats. n = 4-8 rats per group. Scale bar, 40 µm. *p < 0.05, by a unpaired two-tailed Student’s t-test, data represent means ± SEMs.

Table S1. Primer sequences of target genes

|  | Forword (5’→3’) | Reverse (5’→3’) |
| --- | --- | --- |
| IL-1β | AAGATGAAGGGCTGCTTCCAAACC | ATACTGCCTGCCTGAAGCTCTTGT |
| IFN-γ | ATTCATGAGCATCGCCAAGTTC | TGACAGCTGGTGAATCACTCTGA |
| TNF-α | TGATCGGTCCCAACAAGGA | TGCTTGGTGGTTTGCTACGA |
| IL-6 | TGATCGGTCCCAACAAGGA | CCACTTGTTGGCTTATGTT |
| IL-10 | GCTCAGCACTGCTATGTTGC | TTGTCACCCCGGATGGAATG |
| IL-4 | CGTGATGTACCTCCGTGCTT | ATTCACGGTGCAGCTTCTCA |
| Caspase-3 | GGAGCTTGGAACGCGAAGAA | ACACAAGCCCATTTCAGGGT |
| Caspase-9 | CAAGAAGAGCGGTTCCTGGT | CAGAAACAGCATTGGCGACC |
| Bcl-2 | GGATCCAGGATAACGGAGGC | ATGCACCCAGAGTGATGCAG |
| Bax | GGGCCTTTTTGCTACAGGGT | TTCTTGGTGGATGCGTCCTG |
| GAPDH | TCTCTGCTCCTCCCTGTTC | ACACCGACCTTCACCATCT |
